# Prevalence of coccidia in domestic pigs in China between 1980 and 2019: a systematic review and meta-analysis

**DOI:** 10.1186/s13071-021-04611-x

**Published:** 2021-05-10

**Authors:** Qing-Long Gong, Wen-Xuan Zhao, Yan-Chun Wang, Ying Zong, Qi Wang, Yang Yang, Yi Yang, Kun Shi, Jian-Ming Li, Xue Leng, Rui Du, Quan Zhao

**Affiliations:** 1 College of Chinese Medicine Materials, Jilin Agricultural University, Changchun, 130118 Jilin People’s Republic of China; 2 College of Animal Science and Technology, Jilin Agricultural University, Changchun, 130118 Jilin People’s Republic of China; 3 College of Pharmacy, Guizhou University of Traditional Chinese Medicine, Guiyang, 550025 Guizhou People’s Republic of China; 4 School of Life Science, Jilin Normal University, Siping, 136000 Jilin People’s Republic of China; 5 College of Life Science, Changchun Sci-Tech University, Shuangyang, 130600 Jilin People’s Republic of China

**Keywords:** Pigs, Coccidia, Coccidiosis, *Cystoisospora suis*, Prevalence, Meta-analysis

## Abstract

**Background:**

Swine coccidiosis, a protozoan disease caused by coccidia, can result in diarrhoea and weight loss in piglets and even economic losses in the pig industry. Here, we report the first systematic review and meta-analysis of the prevalence of coccidia (including *Eimeria* spp. and *Cystoisospora suis*) in pigs in China.

**Methods:**

Five databases (PubMed, ScienceDirect, Chinese Web of Knowledge, Wanfang, and Chongqing VIP) were searched and 50 studies (46,926 domestic pigs, 22 provinces) ultimately identified pertaining to the prevalence of coccidia infection from 1980 to 2019. We incorporated the effect size using the random-effects model in the “meta” package in R software and conducted univariate and multivariate meta-regression analyses using a mixed-effects model.

**Results:**

The pooled prevalence rate of coccidia in pigs was 21.9%, including the *C. suis* infection rate of 9.1%. The highest prevalence of coccidia (39.6%) was found in northwest China, and this region also presented the lowest prevalence of *C. suis* (4.7%). In the subgroup analysis based on sampling year, the highest prevalence of coccidia was detected in 2001 or earlier (32.6%), whereas the lowest rate was found in 2012 or later (14.3%). An opposite trend was observed for *C. suis* (5.5% in 2000 or earlier* vs* 14.4% in 2000 or later). The prevalence of coccidia in extensive farming systems (29.5%) was higher than that in intensive farming systems (17.3%). In contrast, the point estimate of *C. suis* prevalence was lower in the extensive farming systems (5.1%) than in the intensive farming systems (10.0%), but the difference was not significant (*P* > 0.05). Among the four age categories, the highest total coccidia prevalence (26.2%) was found in finishing pigs, followed by suckling piglets (19.9%), whereas the highest prevalence of *C. suis* (14.9%) was observed in suckling piglets.

**Conclusions:**

Our findings suggest that coccidia infection in Chinese pigs is common, although the prevalence of *C. suis* in pigs does not receive sufficient attention. We recommend the rational use of anticoccidial drugs to avoid drug resistance and the development of preventive and control measures for *C. suis* to reduce the incidence of swine coccidiosis.
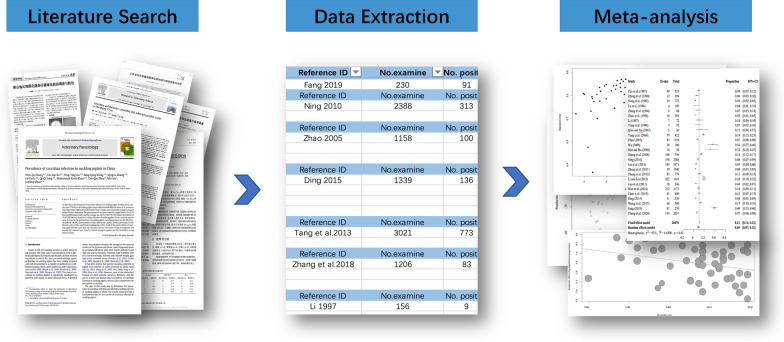

## Highlights

- This study constitutes the first meta-analysis evaluating coccidia prevalence in Chinese pigs.
- Coccidia are prevalent and unevenly distributed in Chinese pig farms.
- The prevalence of coccidia in pigs has decreased in the past 40 years.
- The prevalence of *Cystoisospora suis* appears to have increased in the past 40 years.

## Introduction

Coccidia, a causative agent of coccidiosis, infects various hosts, including livestock, birds and even humans [19, 35]. The pathogenicity of coccidia is affected by many factors, such as the host and the species of coccidia [17, 55].

*Eimeria* and *Cystoisospora* are two genera of coccidia. Thirteen species of *Eimeria* have been identified in pigs, of which eight (*E. debliecki*, Douwes, 1921; *E. neodebliecki*, Vetterling, 1965; *E. perminuta*, Henry, 1931; *E. polita*, Pellérdy, 1949; *E. porci*, Vetterling, 1965; *E. scabra*, Henry, 1931; *E. suis*, Nöller, 1921; and *E. spinosa*, Henry, 1931) are considered to be valid species. Most *Eimeria* spp. are considered to be only mildly pathogenic because they live in superficial epithelial cells [13], athough some (e.g. *E. debliecki*, *E. scabra*, and *E. spinosa*) might cause diarrhoea in piglets [17]. *Eimeria scabra* infection could lead to diarrhoea and anorexia accompanied by signs of nonhemorrhagic (rarely hemorrhagic) enteritis [39, 49]. However, pathogenic infections with *E. debliecki* and *E. spinosa* cannot be easily replicated in experiments due to challenges related to the infectious doses and animal health [21, 38, 48].

Previous studies have identified four species of *Cystoisospora* worldwide (*C. almaataensis*, Paichuk, 1953; *C. neyrai*, Romero-Rodriguez and Lizcano-Herrera, 1971; *C. sundarbanensis*, Ray and Sarkar, 1985; and *C. suis*, Biester and Murray, 1934) [17]. Although *C. suis* was previously named *Isospora suis*, since 2005 it has been re-classified as belonging to the genus *Cystoisospora* [3]. In contrast to *Eimeria* infection, which does not easily induce the development of pathological signs, small doses of *C. suis* can cause clinical disease in neonatal piglets [16]. Specifically, the clinical infection of 7- to 14-day-old piglets with *C. suis* causes their anus to become sticky and their excrement to be yellow or yellow-white with bubbles; 1– 2 days after infection, the excrement resembles water-like loose stools and has a lactic acid odor. *Cystoisospora suis* is therefore considered to be a pathogen of suckling pigs that leads to significant economic losses in the global pig industry [2, 4, 40, 43, 46]. Pigs can also be infected with other coccidia species, but present no clinical signs with such infections [17, 55, 56, 62]. In summary, infection with coccidia might limit the feed intake and inhibit weight gain, thereby lengthening the time to achieve slaughter weight and causing economic losses through coinfection with other diseases [7, 27, 34].

Swine coccidiosis is common in pig herds worldwide. In Brazil and Australia, the prevalence of coccidia in pigs has been reported to be 56.6% (106/187; [6]) and 10.4% (30/289 [18]), respectively. *Cycsoisospora suis* is a common pathogen in suckling piglets, with prevalence rates of 70–90% in Germany, Austria and Switzerland [31] and 78.2% in Spain (229/293).

China is the largest pig-producing country. In 2017, China had 441,588,000 pigs in stock, which corresponded to a pork production of 54,518,000 tons of meat [58]. Coccidia infections are also a serious concern in Chinese pig farms [56]. Therefore, understanding the prevalence of swine infected with coccidia in China and identifying the potential influencing factors are of utmost importance. Here, we report a meta-analysis which we performed with the aim to estimate the prevalence of coccidia (including *Eimeria* spp*.* and *C. suis*) in China and evaluate the potential influencing factors, including geographical location, sampling year, age, sex, feeding model and season.

## Materials and methods

### Search strategy

We conducted this study according to PRISMA guidelines (Additional file 1: Table S1; [28, 29]). Five databases (PubMed, ScienceDirect, Chinese Web of Knowledge, Wanfang, and Chongqing VIP). The restriction information and search strategy details were recorded in Additional file 1: Table S2. The Endnote (X 9.3.1) was used to collate information about all studies. Duplicate studies and reviews were removed according to their titles and abstracts. The inclusion criteria were: (1) the studies used pigs as the research material; (2) the “samples” used in the study were individual samples collected from one pig; (3) the studies reported the prevalence of coccidia; (4) the reported prevalence was calculated based on natural infections; (5) the studies had a cross-sectional design; and (6) the studies were written in English or Chinese.

### Data extraction and quality assessment

Data extraction and recording were independently performed by four trained researchers. Any disagreement or uncertainty regarding the eligibility of a study was further evaluated by the principal author (QLG) of this meta-analysis. We then extracted the following information from each of the included studies: first author, sampling time, sampling location, total number of pigs, numbers of coccidia- and *C. suis*-positive pigs, study design, age and sex of the animals, detection method, feeding model and coccidia species. We generated a database using Microsoft Excel (version 16.35; [32]).

### Quality assessment

The quality of the studies was evaluated based on criteria from the Grading of Recommendations Assessment, Development, and Evaluation methods (GRADE; [11]). Briefly, each of the following items was assigned a score of 1 point if complete information was provided: detection method, sampling year, random sampling, sample collection method and number of subgroups (≥ 4). Thus, all the studies were assigned a score between 0 and 5 points. Studies with a score of 0 or 1 point were considered to be of low quality, whereas those with a score of 2 or 3 points were of moderate quality and those with a score of 4 or 5 points were of high quality [37].

### Statistical analysis

Data were analyzed using the “meta” package [52, 53] in R software version 3.5.2 (R Foundation for Statistical Computing, Vienna, Austria). Data were normalized using logarithmic conversion (PLN), logit transformation “PLOGIT”, arcsine transformation (PAS) and double-arcsine transformation (PFT; [25]). All subsequent analyses were conducted using the transformed proportions as the effect size statistic and the inverse of the variance of the transformed proportions. In this article, we converted the summary proportion and the corresponding confidence interval (CI) back to proportions for ease of interpretation [52]. We used “PLN” for the pooled data (Additional file 1: Table S3; [10, 25]).

In our study, heterogeneity was analyzed according to the *I*^*2*^ statistic, a *χ*^2^-based test, and the Q-test. Due to the obvious heterogeneity in the included studies, we selected the random-effects model to summarize the overall and subgroup estimates [1, 54]. Forest plots were used to visualize the statistical results of the meta-analysis. We tested the publication bias of the studies using Egger’s test, with *P* > 0.05 indicating publication bias. A sensitivity analysis was performed in which one study was removed at a time, and the other studies were used to estimate whether the results were significantly affected by the study that was removed. Subgroup and meta-regression analyses were performed to evaluate the potential sources of heterogeneity, and the factors that caused the observed heterogeneity were assessed through meta-regression. When analyzing the total prevalence of coccidia, we evaluated the geographical region (south China* vs* other regions), detection method (direct smear* vs* other methods), sampling year (2000 or earlier* vs* 2001–2010* vs* 2011 or later), age (finishing pigs* vs* other ages), sex (boars* vs* sows), feeding model (intensive* vs* extensive), season (autumn and winter* vs* spring and summer) and quality level (high* vs* other quality levels). When analyzing the total prevalence of *C. suis*, we investigated the geographical region (north China* vs* other regions), detection method (flotation method with NaCl* vs* other methods), sampling year (2000 or earlier* vs* 2001–2010* vs* 2011 or later), age (suckling piglets* vs* other ages), and feeding model (intensive* vs* extensive). In addition, we used the provinces as a covariate and included each related subgroup in a joint analysis (multivariate meta-regression based on the mixed-effects model) to explain the heterogeneity caused by the different provinces. The R software code used in this meta-analysis is provided in Additional file 1: Table S4.

## Results

### Studies included

**Fig. 1:**
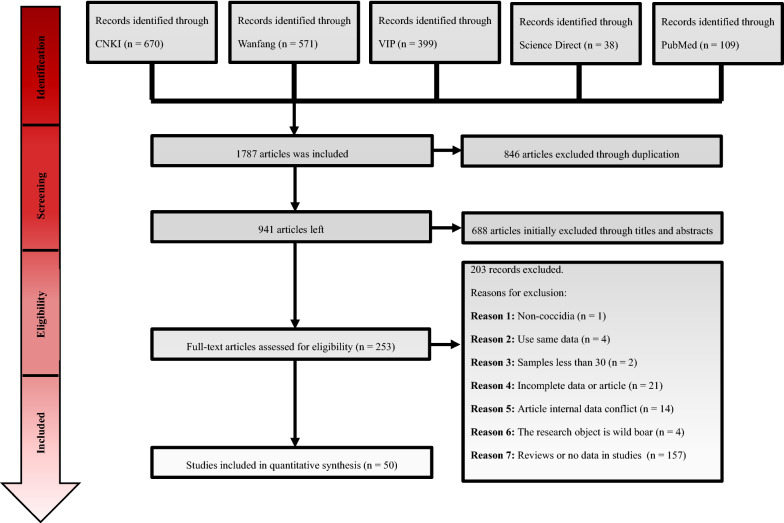
Flow diagram of the literature search and selection of articles to be included in the meta-analysis

The search of the databases identified 1787 records. After removing repeated studies and studies with mismatched abstracts or topics, a total of 253 studies were carefully reviewed, and 50 studies were ultimately included in our meta-analysis (Fig. 1).

### Results of the meta-analysis

**Fig. 2:**
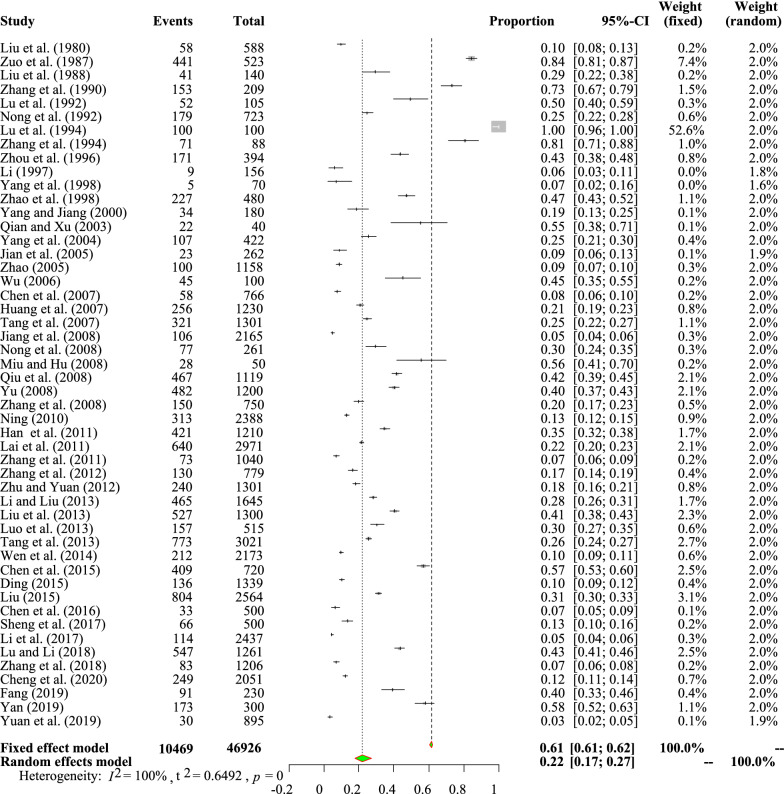
Forest plot of coccidia prevalence in Chinese pigs. The length of the horizontal line represents the 95% confidence interval (*CI*), and the diamond shows the summarized effect.

The studies included in the meta-analysis exhibited high heterogeneity (*I*^*2*^ = 100.00%, *P* *<* 0.01; Fig. 2). In terms of quality (GRADE criteria), five studies were of low quality (0 or 1 point), 25 studies were of moderate quality (2 or 3 points) and 20 studies were of high quality (4 or 5 points; Table 1, Additional file 1: Tables S5, S6).

**Table 1:** Pooled prevalence of coccidia in pigs in China

Variables	Number of studies	Number of pigs tested	Number of pigs positive for coccidia	Percentage pigs positive for coccidia (95% CI)	Heterogeneity			Univariate meta-regression		Joint analysis^a^
					*χ*^2^	*P* value	*I*^2^ (%)	*P* value	Coefficient (95% CI)	Adjusted *R*^2^^b^
Region^c^								0.047	0.426 (0.005–0.847)	29.02%
North China	1	230	91	39.6% (33.7–46.4)	0.00	–	–			
Northwest China	4	1776	394	39.6% (16.7–93.6)	556.35	< 0.01	99.5%			
Northeast China	3	1657	657	33.7% (23.3–48.7)	178.30	< 0.01	96.1%			
East China	14	11132	1876	17.6% (10.7–28.9)	5747.85	0.00	99.7%			
Central China	13	11583	2889	25.0% (18.2–34.2)	1360.95	< 0.01	99.1%			
South China	13	12716	2548	16.1% (12.3–21.0)	791.56	< 0.01	97.9%			
Southwest China	11	7892	2014	26.4% (16.4–42.4)	2155.79	0.00	99.5%			
Detection methods								0.014	1.205 (0.240–2.170)	32.3%
Centrifugal flotation method	4	1701	613	40.7% (19.8–83.6)	479.61	< 0.01	99.4%			
Direct smear	3	3198	174	6.7% (3.0–14.9)	48.28	< 0.01	95.9%			
Flotation method (NaCl)	23	18197	4290	26.8% (18.8–38.1)	8450.65	0.00	99.7%			
Flotation method (NaCl, Suc)	8	9540	2375	24.0% (16.5–34.9)	831.00	0.00	99.2%			
Flotation method (Suc)	3	3439	805	11.3% (4.3–29.7)	48.47	< 0.01	95.9%			
Other methods	8	6905	809	12.3% (7.6–19.1)	340.04	< 0.01	97.9%			
Sampling years								0.002	− 0.538 (− 0.876 to − 0.200)	57.81%
2000 or earlier	14	3756	1540	32.6% (25.8–41.1)	1479.09	< 0.01	98.6%			
2001–2011	16	18208	4021	20.3% (16.1–25.6)	1078.59	< 0.01	98.6%			
2012 or later	9	12381	2061	14.3% (8.2–24.8)	1639.23	0	99.5%			
Pig ages^d^								0.025	− 0.400 (− 0.750 to − 0.049)	43.93%
Suckling piglets	30	13552	2764	19.9% (17.0–23.4)	1189.50	< 0.01	95.9%			
Weaning pigs	20	6224	806	11.6% (8.2–16.4)	621.77	< 0.01	96.8%			
Growing pigs	13	6611	884	12.0% (8.7–16.6)	558.78	< 0.01	96.4%			
Finishing pigs	16	3371	1143	26.2% (20.1–34.1)	601.53	< 0.01	97.3%			
Gender								0.533	0.178 (− 0.382 to 0.739)	7.44%
Boars	13	1565	386	19.4% (13.5–27.9)	188.80	< 0.01	93.6%			
Sows	19	4449	1075	21.1% (14.8–30.3)	1409.60	< 0.01	98.7%			
Feeding model								0.016	0.529 (0.100–0.958)	40.2%
Extensive	14	7014	1788	29.5% (18.4–47.1)	3766.53	0.00	99.7%			
Intensive	36	35564	7224	17.3% (14.4–20.9)	3235.66	0.00	98.9%			
Season^e^								0.018	0.610 (0.103–1.116)	47.11%
Spring and summer	11	4431	1383	32.0% (24.0–42.7)	716.32	< 0.01	97.9%			
Autumn and winter	5	2554	522	16.0% (9.6–26.5)	579.36	< 0.01	97.6%			
Quality level								0.000	0.741 (0.352 –1.131)	28.84%
Low	5	4163	1468	32.9% (26.1–41.6)	108.57	< 0.01	96.3%			
Middle	25	18109	4998	29.1% (22.1–38.3)	8028.51	< 0.01	99.7%			
High	20	24654	4003	14.1% (10.4–19.0)	2255.46	< 0.01	99.2%			
Total	50	46926	10469	21.9% (17.5–27.4)	17697.68	0.000	99.7%			

*CI* Confidence interval

^a^Joint analysis: results of each subgroup joint test with provinces

^b^Adjusted *R*^2^: proportion of between-study variance explained

^c^Northern China: Hebei; Northwestern China: Shaanxi. Northeastern China: Heilongjiang, Jilin, Liaoning. Eastern China: Anhui, Fujian, Jiangsu, Jiangxi, Shandong, Shanghai, Zhejiang. Central China: Henan, Hubei. Southern China: Guangdong, Guangxi, Hainan, Hubei, Hunan. Southwestern China: Chongqing, Sichuan, Yunnan

^d^Suckling piglets (age: days 0–30); weaning pigs (days 28–63); growing pigs (days 63–168); finishing pigs (older than 168 days)

^e^Spring and summer: March through August. Autumn and winter: September through February.

The funnel chart graphic was asymmetric, which revealed the existence of publication bias or small sample size bias in the studies (Additional file 2: Figure S1). The publication bias was further tested using Egger’s test (*P* < 0.05, Additional file 1: Table S7, Additional file 2: Figure S2), and the results revealed the existence of publication bias. The sensitivity analysis showed that the pooled data obtained after individual studies were excluded did not notably change the result, which indicated the reliability of our results (Additional file 2: Figure S3).

#### Meta-analysis of coccidia infection in pigs in China

**Fig. 3:**
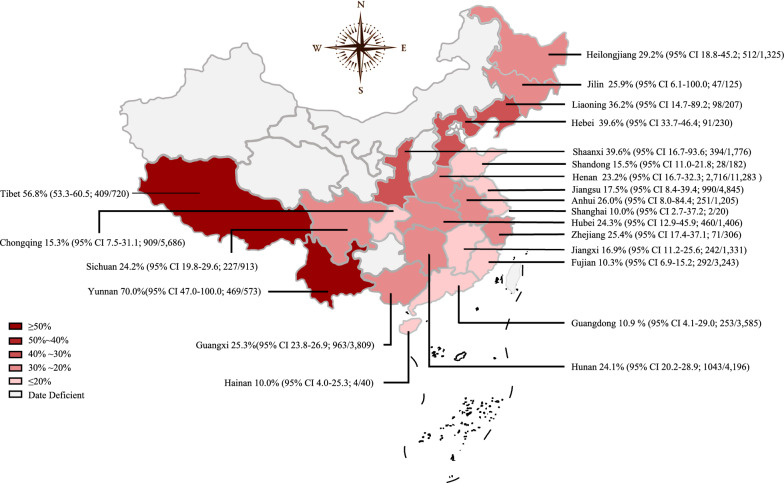
Map of coccidia prevalence in pigs in China.* CI* Confidence interval

Our meta-analysis included studies carried out in 22 provinces in seven regions of China (Table 1; Fig. 3; Additional file 1: Table S5). The pooled prevalence of coccidia in pigs was 21.9% (95% CI 17.5–27.4%; 10,469/46,926 pigs), and the highest and lowest prevalences were obtained in borthwest China (39.6%, 95% CI 16.7–93.6%; 394/1776) and south China (16.1%, 95% CI 12.3–21.0%; 2548/12,716; Table 1). More specifically, the highest prevalence of coccidia infections was observed in Yunnan Province (70.0%, 95% CI 47.0–100%; 469/573) and Tibet (56.8%, 95% CI 53.3–60.5%; 409/720; Fig. 3).

The pooled prevalence of coccidia infection detected in 2000 or earlier was 32.6% (95% CI 25.8–41.1%; 1540/3756), and this prevalence was higher (*P* *<* 0.05) than that obtained in the other tested time periods. The analysis of various detection methods revealed that the highest prevalence (*P* *<* 0.05) was obtained with the centrifugal flotation method (40.7%, 95% CI 19.8–83.6; 613/1701). In the age subgroup analysis, the coccidia prevalence rates in suckling piglets (19.9%, 95% CI 17.0–23.4%; 2764/13552) and finishing pigs (26.2%, 95% CI 20.1–34.1%; 1143/3371) were higher than those in the other two age categories. Coccidia prevalence was lower in boars (19.4%, 95% CI 13.5–27.9%; 386/1565) than in sows (21.1%, 95% CI 14.8–30.3%; 1075/4449), but the difference was not significant (*P* > 0.05). As shown in Table 1, a higher (*P* *<* 0.05) coccidia prevalence was obtained in extensive pig farming systems (29.5%, 95% CI 18.4–47.1%; 1788/7014) than in intensive pig farming sysems (17.3%, 95% CI 14.4–20.9%; 7224/35,564). In the season subgroup analysis, the pooled prevalence of coccidia in pigs was significantly higher (*P* *<* 0.05) in the spring and summer (32.0%, 95% CI 24.0–42.7%; 1383/4431) than in the autumn and winter (16.0%, 95% CI 9.6–26.5%; 522/2554). We identified 11 species of coccidia, and the prevalence of *E. spinosa* (8.6%, 95% CI 5.2–14.2%; 272/2362) was lower than those of other species (Additional file 1: Table S8) (authors’ remark: although it might not currently be a confirmed species, *E. yanglingensis* was included in our study). The univariate meta-regression results revealed that sex (*P* > 0.05) was not the main source of the observed heterogeneity (Table 1; Additional file 1: Table S9).

Based on our calculated prevalence of coccidia (21.9%, 95% CI 17.5–27.4%; 10,469/46,926), we used data from the “Chinese Animal Husbandry and Veterinary Yearbook” published in 2018 to determine that 96,707,772 (77,277,900–120,995,112) pigs in China were infected with coccidia in 2017 (Table 2).

**Table 2:** Estimates of coccidia infection in pigs in China

Region	Estimated number of pigs in various regions of China^a^	Prevalence of Coccidia infection of pigs in various regions of China (95% CI)	Estimated number of pigs with Coccidia infection
North China	32,997,000	39.6% (33.7–46.4)	13,066,812 (11,119,989–15,310,608)
Northeast China	36,530,000	33.7% (23.3–48.7)	12,310,610 (8,511,490–17,790,110)
East China	92,947,000	17.6% (10.7–28.9)	16,358,672 (9,945,329–26,861,683)
Central China	109,366,000	25.0% (18.2–34.2)	27,341,500 (19,904,612–37,403,172)
South China	48,261,000	16.1% (12.3–21.0)	7,770,021 (5,936,103–10,134,810)
Southwest China	102,366,000	26.4% (16.4–42.4)	27,024,624 (16,788,024–43,403,184)
Northwest China	19,121,000	39.6% (16.7–93.6)	7,571,916 (3,193,207–17,897,256)
Total	441,588,000	21.9% (17.5–27.4)	96,707,772 (77,277,900–120,995,112)

The values in parentheses in column 4 was defined as the product of the estimated value (column 2) and the upper and lower bounds of the confidence interval (column 3)

^a^Estimates of the number of pigs in each region were obtained from 2017 data of the Chinese Animal Husbandry and Veterinary Yearbook report

#### Meta-analysis of *C. suis* infection in pigs in China

**Fig. 4:**
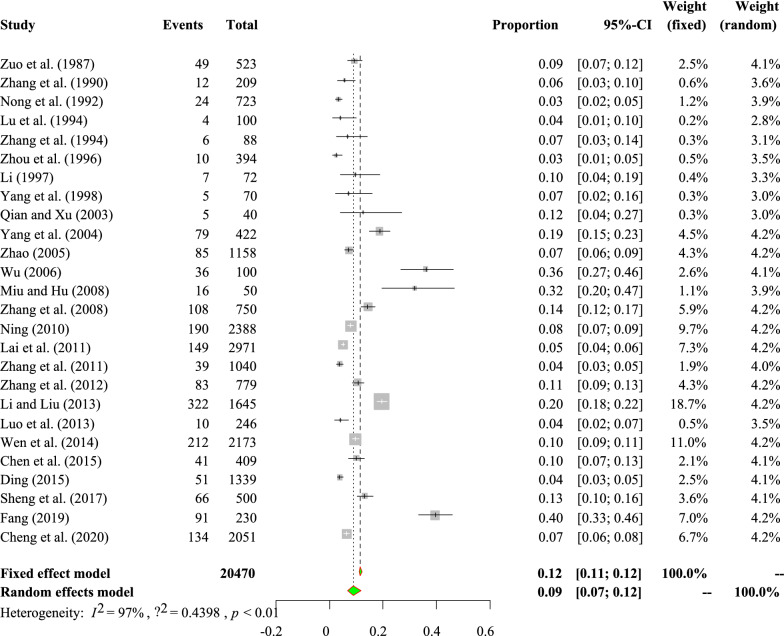
Forest plot of *Cystoisospora suis* prevalence in Chinese pigs. The length of the horizontal line represents the 95% confidence interval, and the diamond shows the summarized effect.

The pooled prevalence of *C. suis* infection in pigs in China was 9.1% (95% CI 6.9–11.9; 1834/20,470; Table 3; Fig. 4; Additional file 1: Table S8). The analysis of regional subgroups revealed that the lowest *C. suis* prevalence was in northwest China (4.7%, 95% CI 3.3–6.7; 69/1636). The detection method that yielded the highest detection rate was the flotation method (with sucrose; 56%, 95% CI 43.8–71.6; 28/50). A higher prevalence was detected in the samples obtained in 2012 or later (14.4%, 95% CI 8.6–24.3; 659/5363) than in the samples collected at the other tested time periods. Among the four age categories, suckling piglets presented the highest (*P* *<* 0.05) rate of *C. suis* infection (14.9%, 95% CI 10.9–20.4; 1066/7072) (Table 3). The univariate meta-regression analyses of *C. suis* prevalence identified “region,” “sampling years” and “pig ages” as sources of heterogeneity (Table 3).

**Table 3:** Pooled prevalence of *Cystoisospora suis* in pigs in China

Variables	Number of studies	Number of pigs tested	Number of pgs positive for *C. suis*	Percentage pigs positive for *C. suis* (95% CI)	Heterogeneity			Univariate meta-regression		Joint analysis
					*χ*^2^	*P* value	*I*^2^ (%)	*P* value	Coefficient (95% CI)	Adjusted *R*^2^
Region^a^								0.013	− 1.542 (− 2.757 to − 0.327)	56.16%
North China	1	230	91	39.6% (33.7–46.4)	0.00	–	–			
Northwest China	3	1636	69	4.7% (3.3–6.7)	3.21	< 0.01	37.7%			
East China	6	3953	362	10.2% (5.6–18.6)	136.20	0.00	96.3%			
Central China	5	6741	527	7.3% (5.2–10.4)	62.66	< 0.01	93.6%			
South China	4	2510	358	8.3% (2.9–23.9)	80.55	< 0.01	96.3%			
Southwest China	6	4621	344	10.3% (5.7–18.6)	150.67	0.00	96.7%			
Detection methods								0.026	0.631 (0.076–1.187)	22.22%
Centrifugal flotation method	4	1701	110	9.4% (3.8–23.3)	69.28		95.7%			
Flotation method (NaCl)	11	8020	651	7.1% (4.6–11.1)	259.96	0.00	96.2%			
Flotation method (NaCl, Suc)	7	6665	860	13.9% (8.8–22.2)	324.16		98.1%			
Flotation method (Suc)	1	50	28	56% (43.8–71.6)	0.00	–	–			
Others	2	2673	278	11.2% (8.3–15.0)	5.25	0.00	81.0%			
Sampling years								0.003	0.842 (0.291–1.394)	54.24%
2000 or earlier	8	2179	117	5.5% (3.7–8.3)	29.70	< 0.01	76.4%			
2001 to 2011	10	11303	1107	12.1% (8.1–18.0)	441.98	< 0.01	98.0%			
2012 or later	5	5363	659	14.4% (8.6–24.3)	204.10	0	98.0%			
Pig ages								0.001	− 0.975 (− 1.418 to − 0.532)	51.36%
Suckling piglets	14	7072	1006	14.9% (10.9–20.4)	586.99	< 0.01	96.6%			
Weaning pigs	9	3286	202	5.5% (3.5–8.7)	73.97	< 0.01	89.2%			
Growing pigs	6	2441	133	5.8% (3.7–9.0)	33.34	< 0.01	82.0%			
Finishing pigs	2	528	32	6.3% (4.1–9.6)	2.72	< 0.01	26.4%			
Feeding model								0.081	0.722 (0.021–1.533)	34.43%
Extensive	4	3202	223	5.1% (3.0–8.5)	15.35	< 0.01	80.5%			
Intensive	14	10991	1145	10.0% (6.9–14.6)	562.01	< 0.01	97.7%			
Total	26	20470	1834	9.1% (6.9–11.9)	885.02	0.000	97.2%			

^a^Northern China: Hebei; Northwestern China: Shaanxi. Northeastern China: Heilongjiang, Jilin, Liaoning. Eastern China: Anhui, Fujian, Jiangsu, Jiangxi, Shandong, Shanghai, Zhejiang. Central China: Henan, Hubei. Southern China: Guangdong, Guangxi, Hainan, Hubei, Hunan. Southwestern China: Chongqing, Sichuan, Yunnan

The results from the subsequent joint analysis showed that provinces could explain 7.44–57.81% of the heterogeneity in each subgroup (Tables 1, 3).

## Discussion

Pork is an important source of protein for the increasing world population. Coccidia infection affects animal growth, reduces production performance and leads to coinfections with other diseases, such as rotavirus, *Escherichia coli*, transmissible gastroenteritis viruses and clostridia [7, 8, 30, 59]. Among the various coccidia, *C. suis* is highly pathogenic to piglets [17]. We reported here the first systematic review and meta-analysis carried out on the prevalence of coccidia and *C. suis* in pigs in China. In this meta-analysis, we investigated the pooled prevalence of coccidia in pigs over the past 40 years and identified 11 coccidia species in China. Based on the results, *E. neodebliecki*, *E. suis* and *E. scabra* may be the main coccidia species in pigs in China, and three species of *Eimeria* may cause diarrhoea in piglets (*E. debliecki*, 16.8%; *E. scabra*, 18.9%; *E. spinosa*, 8.6%). These findings indicate that the economic losses caused by *Eimeria* spp. should not be ignored in the Chinese pig breeding industry. Notably, the prevalence of *C. suis* in pigs in China (9.1%) is markedly lower than the overall estimate of coccidia, and we speculate that the high prevalence of *Eimeria* spp. is responsible for this. Unfortunately, the prevalences of *Eimeria* spp. are barely mentioned in the studies included in our meta-analysis, and we therefore cannot clearly calculate the pooled prevalence of *Eimeria* spp. alone. In the region, sampling year and feeding model subgroup analyses, the pooled estimates obtained for *C. suis* were the opposite of those found for coccidia, leading us to infer that farmers may ignore the following important fact when attempting to prevent and control coccidia: *C. suis* is more environmentally resistant than *Eimeria* spp*.* [9, 17, 45]. Anticoccidial drug resistance has also been reported [44]. Therefore, the development of preventive and control measures is important for reducing the prevalence of *C. suis* in pigs in China.

The prevalence of coccidia in the northwest and northeast regions of China was higher than the overall pooled estimate, and the lowest prevalence was found in the region of southern China. Our analysis also revealed that the lowest prevalence of *C. suis* was in northwest China. According to the Chinese Animal Husbandry and Veterinary Yearbook 2018, 214 and 627 farms produced ≥ 5000 pigs per year in northwest and northeast China, respectively, and these are the areas with the fewest number of large pig farms (< 1000) among the seven regions in China [58]. This finding could be attributed to the technology and scale of the local pig feeding models. We also used feeding models and provinces as covariates to study relationships and found that provinces can explain 40.23% of the heterogeneity of the feeding model subgroup (*R*^2^ = 40.23%), which implies that different provinces have different scales of farming that regional differences are one of the reasons for the heterogeneity of farming mode subgroups. Only one study from northern China was included, and no study on the prevalence of *C. suis* in northeast China was included. Therefore, the investigation of *C. suis* should be strengthened to determine the true prevalence of *C. suis* in each region.

The highest prevalence of coccidia in pigs was obtained in Yunnan and Tibet, and the lowest prevalence was observed in Hainan, Shanghai and Fujian. We speculated that the lower prevalence in these southern provinces or cities might be related to their economic and breeding conditions. The number of free-range pig farms is relatively higher in less developed areas. The understanding of pig diseases by farmers also showed marked variation, and thus achieving improved breeding conditions and disease prevention in these areas is difficult [51, 57]. In China, the regionalization of pig breeding is obvious. Due to feed resources, labor resources and consumer markets, pig breeding is mainly concentrated along the coast of the Yangtze River, the north China coast and some major grain-producing areas, including Sichuan, Henan, Hunan, Shandong, Hubei, Guangdong, Hebei, Yunnan, Guangxi and Jiangxi. These provinces are the top ten pig-breeding areas in China [5]. People in Ningxia, Qinghai and certain other places seldom eat pork due to their religious beliefs, and the pig-breeding industry in these places is thus underdeveloped [24]. Therefore, research on the prevalence of coccidia in pigs in these areas might be insufficient. Additionally, we could not accurately determine the prevalence rates of coccidia in pigs in the relevant provinces. However, we believe that the pooled estimates obtained in this study reflect the prevalence rates of coccidia in China. Moreover, coccidia infections in China show regional diversity [60].

In the season subgroups, the prevalence of coccidia in the spring and summer was twofold higher than that in autumn and winter, which indicates that coccidia infection is related to temperature and humidity. Coccidia infection might occur throughout the year, but the incidence of coccidia in pigs is significantly higher under conditions of high temperature and high rainfall [12, 42]. Based on these findings, deworming procedures should be strengthened during the spring and summer.

The subgroup analysis based on the detection method revealed that the direct smear and centrifugal flotation methods had the lowest and highest detection rates, respectively. Although the direct smear method involves an easily implemented protocol, the detection of oocysts might be difficult due to interference by lipids and other impurities [15, 33]. The flotation method, which is commonly used to test for intestinal parasites, relies on a liquid with a large specific gravity to float the protozoan oocysts and collect them on the surface of a separation medium. Most of the studies included in this meta-analysis used the flotation method (with NaCl). In general, the use of saturated sugar or sugar–salt solutions could reduce the number of lipid bubbles in the counting chamber [14, 17]. However, NaCl is more readily available than other media. The medium used in the centrifugal flotation method is usually ZnSO_4_ because ZnSO_4_ can reduce lipid interference during centrifugation, but this medium is more costly than NaCl or sucrose. Henriksen and Christensen suggested the use of a saturated sugar solution instead of saturated sodium chloride for the detection of *C. suis* [14]. However, the flotation method (with sucrose) is mostly used for the detection of *Cryptosporidium* oocysts in feces. Our research found that the flotation method (with NaCl and sucrose) might be more suitable for the detection of *C. suis* than the other methods. In general, molecular biological testing methods (e.g., PCR, nested PCR, RT-PCR and other nucleic acid detection methods not mentioned in the included studies) are more sensitive. Unfortunately, these methods require trained professionals and are relatively expensive. In contrast, the low number of studies on molecular techniques among the studies included in our meta-analysis could be attributed to the fact that researchers in China ignore coccidiosis in pigs. This neglect can also be reflected by the lack of reports on coccidia in some provinces. In addition, because piglets infected with *C. suis* usually excrete oocysts very rapidly, accurate timing and a sufficiently sensitive detection method are important for a correct diagnosis. The repeated testing of piglets might solve this problem to a certain extent. However, we were unable to quantify the sampling frequency because almost none of the included studies provided this information.

China joined the World Trade Organization (WTO) in 2001 [47] and issued a mid-to-long-term animal disease prevention plan (2012–2020) in 2012 that strengthens their measures for animal disease prevention and control [50]. Therefore, we selected 2001 and 2012 as the time points for this study on the prevalence of coccidia and *C. suis* in China. The findings revealed that the prevalence of coccidia in Chinese pig farms has been decreasing over the past 40 years, as determined by multifactor meta-regression (Additional file 2: Figure S4). The animal disease prevention and control policy has been gradually implemented and has played a positive role in the control of coccidia. Unfortunately, only *Eimeria* appears to be under control because the prevalence of *C. suis* has increased in the past 40 years (Additional file 2: Figure S5), which could be attributed its greater environmental exposure and drug resistance.

Intensive farming aids the development and implementation of a more standardized chemotherapeutic process for controlling coccidiosis, which could improve the breeding environment and reduce the probability that pigs come into contact with external pathogens [63]. However, an interesting outcome of this meta-analysis was obtained with the feeding mode subanalysis: intensive farming was associated with increased *C. suis* infections (*P <* 0.05). In China, toltrazuril is commonly used in pig farms to control *C. suis*. This drug has a low toxicity and can be used at various stages of *C. suis* development. However, the abuse and misuse of this drug contributes to drug resistance and thereby reduces the effectiveness of prevention and control measures [36]. Monitoring the control effect of toltrazuril and following the instructions of veterinarians regarding its proper use are important for avoiding drug resistance. Effective (cresol-type) disinfectants against coccidia can reduce the number of oocysts in the environment. In addition, we found that the prevalence of *C. suis* in intensive farming was twofold higher than that in systems of free-range breeding. In the past 40 years, China has become the world’s largest pig-raising country. Small-scale and free-range farms in both poor and developed regions are gradually being phased out and replaced by large-scale farms [26, 61], which have a faster production cycle and a higher breeding density. These trends may account for a proportion of the increased prevalence of *C. suis*. Separate detection and control methods for *C. suis* should be used to reduce economic losses. Notably, some of the studies, particularly those conducted in earlier years, did not investigate and control for the prevalence of *C. suis* in pigs, which might have led to our ignoring the true prevalence of *C. suis*. In contrast, the lack of studies in the extensive group might also have affected our results.

Coccidia infections may occur throughout the lifetime of pigs. Adult pigs might have more opportunities to come into contact with oocysts than young animals; however, piglets aged 5–50 days are reported to be the most susceptible group [22, 23], which is consistent with our findings. The lower *C. suis* prevalence rates in older pigs might be strongly related to age [20]. Finishing pigs need a clean breeding environment to reduce their contact with coccidia oocysts. Our research revealed that the point estimates of coccidia prevalence were slightly higher in sows than in boars [17, 42], but the differences were not significant (*P <* 0.05), as reported elsewhere [42]. Schwarz et al. noted that sows might confer piglet resistance to *C. suis* through lactation [41]; however, another study pointed out that coccidiosis infection in piglets might originate from oocysts introduced into the farrowing bed by the sow [59]. Therefore, the internal relationship between sows carrying *C. suis* and *C. suis* infection in piglets needs further research and might play an important role in the prevention of piglet *C. suis* infection.

Most of the studies included in our meta-analysis were of moderate quality. Nineteen studies did not include “four or more subgroups,” which suggests that researchers should investigate more potential risk factors to clarify the specific cause of coccidia infection and provide a scientific basis for coccidiosis control.

Our meta-analysis has some limitations. Firstly, we attempted to identify all studies related to coccidia in pigs through searches using several different MeSH terms; however, these searches might not have detected all relevant studies. Second, some of the subgroups had insufficient data, which might have affected our results; thus, the assessment of risk factors leading to coccidia infection in pigs should be strengthened. Third, some studies did not separately detect the prevalence of *C. suis*; therefore, the data on *C. suis* might not be sufficient. Fourth, the information on suckling piglets was not sufficient; therefore, the most susceptible age for coccidia (particularly *C. suis*) in suckling piglets could not be determined. Fifth, we were unable to quantify the sampling frequency because almost none of the included studies provided this information. Sixth, the included studies hardly mentioned the prevalence of *Eimeria* spp., and we were thus unable to present the pooled prevalence of *Eimeria* spp. alone. We also restricted the language to English and Chinese, and as a result, related articles in other languages might not have being retrieved.

In conclusion, coccidia prevalence has decreased over the past 40 years, whereas *C. suis* prevalence has increased during this same time frame. To prevent and control the spread of coccidia, particularly *C. suis*, in pigs, further epidemiological surveillance and comprehensive prevention and control programs are needed. Adjusting the farming model, improving the feeding environment and animal welfare, administering reasonable medications and developing prevention plans for *C. suis* might help alleviate the current situation.

## Supplementary Information

**Additional file 1: Table S1.** PRISMA Checklist item. **Table S2.** Detailed search strategy and restrictions. **Table S3.** Normal distribution test for the normal rate and the different conversion of the normal rate. **Table S4.** The code in R for this meta-analysis. **Table S5**. Studies included in the analysis. **Table S6.** Included studies and quality scores. **Table S7.** Egger’s for Publication Bias. **Table S8.** Pooled prevalence of different coccidia species in China. **Table S9.** List of abbreviations.

**Additional file 2: Figure S1.** Funnel plot with pseudo 95% confidence intervals used in the assessment of publication bias. **Figure S2.** Assessment of publication bias using Egger’s test. **Figure S3.** Sensitivity analysis. **Figure S4.** Multivariate meta-regression analysis by publication year. Funnel plot with pseudo 95% confidence intervals used in the assessment of publication bias (*C. suis*). Assessment of publication bias (*C. suis*) using Egger’s test. Sensitivity analysis (*C. suis*). **Figure S5.** Multivariate meta-regression analysis by publication year (*C. suis*).

## Data Availability

The datasets used and/or analysed during the current study are available from the corresponding author upon reasonable request.
