# Supplementary material for: Prevalence of coccidia in domestic pigs in China between 1980 and 2019: a systematic review and meta-analysis
Source: Parasit Vectors. 2021 May 10;14:248. doi: 10.1186/s13071-021-04611-x (PMC8108339; doi:10.1186/s13071-021-04611-x)
Supplement: Supplementary file 1 — Additional file 1: Table S1. PRISMA Checklist item. Table S2. Detailed search strategy and restrictions. Table S3. Normal distribution test for the normal rate and the different conversion of the normal rate. Table S4. The code in R for this meta-analysis. Table S5. Studies included in the analysis. Table S6. Included studies and quality scores. Table S7. Egger’s for Publication Bias. Table S8. Pooled prevalence of different coccidia species in China. Table S9. List of abbreviations. [file 13071_2021_4611_MOESM1_ESM.docx]

**Table S1.** PRISMA Checklist item.

| **Section/topic** | **#** | **Checklist item** | **Reported on page #** |
| --- | --- | --- | --- |
| **TITLE** |  |  |  |
| Title | 1 | Prevalence of coccidia in domestic pigs from mainland China between 1980 and 2019: A systematic review and meta-analysis | 1 |
| **ABSTRACT** |  |  |  |
| Structured summary | 2 | Provide a structured summary including, as applicable: background; objectives; data sources; study eligibility criteria, participants, and interventions; study appraisal and synthesis methods; results; limitations; conclusions and implications of key findings; systematic review registration number. | 2-3 |
| **INTRODUCTION** |  |  |  |
| Rationale | 3 | Describe the rationale for the review in the context of what is already known. | 4-5 |
| Objectives | 4 | Provide an explicit statement of questions being addressed with reference to participants, interventions, comparisons, outcomes, and study design (PICOS). | 4-5 |
| **METHODS** |  |  |  |
| Eligibility criteria | 5 | Specify study characteristics (e.g., PICOS, length of follow-up) and report characteristics (e.g., years considered, language, publication status) used as criteria for eligibility, giving rationale. | 5-6 |
| Information sources | 6 | Describe all information sources (e.g., databases with dates of coverage, contact with study authors to identify additional studies) in the search and date last searched. | 5-7 |
| Search | 7 | Present full electronic search strategy for at least one database, including any limits used, such that it could be repeated. | 5-7 |
| Study selection | 8 | State the process for selecting studies (i.e., screening, eligibility, included in systematic review, and, if applicable, included in the meta-analysis). | 7-8 |
| Data collection process | 9 | Describe method of data extraction from reports (e.g., piloted forms, independently, in duplicate) and any processes for obtaining and confirming data from investigators. | 7-8 |
| Data items | 10 | List and define all variables for which data were sought (e.g., PICOS, funding sources) and any assumptions and simplifications made. | 7-8 |
| Risk of bias in individual studies | 11 | Describe methods used for assessing risk of bias of individual studies (including specification of whether this was done at the study or outcome level), and how this information is to be used in any data synthesis. | 8-9 |
| Summary measures | 12 | State the principal summary measures (e.g., risk ratio, difference in means). | 8-9 |
| Synthesis of results | 13 | Describe the methods of handling data and combining results of studies, if done, including measures of consistency (e.g., I^2^) for each meta-analysis. | 8-9 |
| Risk of bias across studies | 14 | Specify any assessment of risk of bias that may affect the cumulative evidence (e.g., publication bias, selective reporting within studies). | 8-9 |
| Additional analyses | 15 | Describe methods of additional analyses (e.g., sensitivity or subgroup analyses, meta-regression), if done, indicating which were pre-specified. | 8-9 |
| **RESULTS** |  |  |  |
| Study selection | 16 | Give numbers of studies screened, assessed for eligibility, and included in the review, with reasons for exclusions at each stage, ideally with a flow diagram. | 9-10, Figure 1 |
| Study characteristics | 17 | For each study, present characteristics for which data were extracted (e.g., study size, PICOS, follow-up period) and provide the citations. | 9-10, Table S3 |
| Risk of bias within studies | 18 | Present data on risk of bias of each study and, if available, any outcome level assessment (see item 12). | 9-10, Figures 3 and 4, Table 1, Table S4 |
| Results of individual studies | 19 | For all outcomes considered (benefits or harms), present, for each study: (a) simple summary data for each intervention group (b) effect estimates and confidence intervals, ideally with a forest plot. | 9-10, Figures 2 and 6 |
| Synthesis of results | 20 | Present results of each meta-analysis done, including confidence intervals and measures of consistency. | 9-10, Tables 3, 4, and 6 |
| Risk of bias across studies | 21 | Present results of any assessment of risk of bias across studies (see Item 15). | 9-10, Figures S1-S8 |
| Additional analysis | 22 | Give results of additional analyses, if done (e.g., sensitivity or subgroup analyses, meta-regression [see Item 16]). | 9-11, Figure 6, Tables 3, 4, 5, and 6. |
| **DISCUSSION** |  |  |  |
| Summary of evidence | 23 | Summarize the main findings including the strength of evidence for each main outcome; consider their relevance to key groups (e.g., healthcare providers, users, and policy makers). | 12-16 |
| Limitations | 24 | Discuss limitations at study and outcome level (e.g., risk of bias), and at review-level (e.g., incomplete retrieval of identified research, reporting bias). | 16-17 |
| Conclusions | 25 | Provide a general interpretation of the results in the context of other evidence, and implications for future research. | 17-18 |
| **FUNDING** |  |  |  |
| Funding | 26 | Describe sources of funding for the systematic review and other support (e.g., supply of data); role of founders for the systematic review. | 18 |

*From:* Moher D, Liberati A, Tetzlaff J, Altman DG, The PRISMA Group (2009). Preferred Reporting Items for Systematic Reviews and Meta-Analyses: The PRISMA Statement. PLoS Med 6(6): e1000097. doi:10.1371/journal.pmed1000097

For more information, visit: **www.prisma-statement.org**.

**Table S2.** Detailed search strategy and restrictions.

| Database | Limitation | Search formula |
| --- | --- | --- |
| PubMed | All files | *("Coccidia"[Mesh] OR Coccidias OR Coccidium OR Isospora OR Cystoisospora OR Eimeria OR Eimerias) AND ("Sus scrofa"[Mesh] OR Sus scrofa domestica OR Pig, Domestic OR Domestic Pig OR Domestic Pigs OR Pigs, Domestic OR Pigs OR Swine) AND ("China"[Mesh] OR People's Republic of China OR Mainland China OR Manchuria OR Sinkiang OR Inner Mongolia) |
| ScienceDirect | Keywords | “swine”, “pig”, “coccidia”, “Eimeria”, “Isospora”, and “China” |
| CNKI | Advanced Search & Subject term & Fuzzy retrieval and synonym extension | “pig” (Chinese) and “coccidia” (Chinese) |
| Chongqing VIP | Advanced Search & Title or keyword & Fuzzy retrieval and synonym extension | “pig” (Chinese) and “coccidia” (Chinese) |
| WanFang | Advanced Search & Title or keyword & Fuzzy retrieval and synonym extension | “pig” (Chinese) and “coccidia” (Chinese) |

* In PubMed, the Boolean operator “AND” was used to connect theme words, and “OR” was used to connect free words. The theme words were "Coccidia"[Mesh], “*Sus scrofa*” [Mesh], and “China” [Mesh]; the other left words in search formula of PubMed were free words of their theme words.

**Table S3.** Normal distribution test for the normal rate and the different conversion of the normal rate.

| Conversion form | *W* | *P* |
| --- | --- | --- |
| PRAW | 0.897 | 0.000 |
| PLN | 0.964 | 0.134 |
| PLOGIT | NaN | NA |
| PAS | 0.924 | 0.003 |
| PFT | 0.921 | 0.003 |

“PRAW”: original rate; “PLN”: logarithmic conversion; “PLOGIT”: logit transformation; “PAS”: arcsine transformation; “PFT”: double-arcsine transformation; “NaN”: meaningless number; “NA”: missing data.

**Table S4.** The code in R for this meta-analysis.

| Logarithmic conversion (PNL) | rate<-transform [m1, log=log(event/n)];  shapiro.test(rate$log) |
| --- | --- |
| Logit transformation (PLOGIT) | rate<-transform{m1, logit=log[(event/n)/(1-event/n)]};  shapiro.test(rate$logit) |
| Arcsine transformation (PAS) | rate<-transform{m1, arcsin.size=asin[sqrt(event/(n+1))]};  shapiro.test(rate$arcsin) |
| Double-arcsine transformation (PFT) | rate<-transform{m1,darcsin=0.5*[asin(sqrt(event/(n+1)))+asin((sqrt(event+1)/(n+1)))]};  shapiro.test(rate$darcsin) |
| No transformation (PRAW) | rate<-transform[m1, r= event/n];  shapiro.test(rate$r) |

| Forest plots | forest [meta1, xlim=c(-0.2, 0.8)] |
| --- | --- |
| Funnel chart | funnel (meta1) |
| Egger's test | metabias (meta1, method="linreg") |
| The sensitivity analysis | metainf (meta1, pooled = "random") forest (metainf (meta1, pooled = "random"), xlim=c(0, 0.3)) |
| Subgroup analysis | meta1<-metaprop(event, n, study, data=rate, sm="PLN", incr=0.5, allincr=TRUE, addincr=FALSE, title="", byvar= subgroup title, print.byvar=TRUE) |
| Meta-regression analysis | metareg (meta1, ~covariate title) |

**Table S5**. Studies included in the analysis.

| Study ID | Sampling time | Province | Detection method | Positive samples/  total samples (*Coccidia*) | Positive samples/  total samples (*C. suis*) | Quality score | Quality level |
| --- | --- | --- | --- | --- | --- | --- | --- |
| **Central China** |  |  |  |  |  |  |  |
| Liu et al. (1988) | 1985.03-1985.06 | Henan | Others (centrifugal precipitation) | 41/140 | ND* | 1 | Low |
| Zhou et al. (1996) | UN* | Henan | Flotation method (NaCl)* | 102/403 | 10/394 | 3 | Middle |
| Jian et al. (2005) | 2003.03-2003.06 | Henan | Flotation method (Suc)* | 23/262 | ND | 4 | High |
| Zhao (2005) | 2005.04 | Henan | Flotation method (NaCl, Suc)* | 100/1158 | 85/1158 | 4 | High |
| Qiu et al. (2008) | 2006.08-2007.07 | Henan | UN | 467/1119 | ND | 4 | High |
| Zhang et al. (2008) | 2006.11-2007.02 | Henan | Flotation method (NaCl, Suc) | 150/750 | 108/750 | 4 | High |
| Ning (2010) | 2005.05-2008.08 | Henan | Flotation method (NaCl) | 313/2388 | 190/2388 | 4 | High |
| Han et al. (2011) | UN | Henan | UN | 421/1210 | ND | 3 | Middle |
| Zhang et al. (2012) | 2009.09-2010.12 | Henan | Flotation method (NaCl) | 29/151 | ND | 5 | High |
| Liu and Wang (2013) | 2010.05-2011.05 | Henan | UN | 527/1300 | ND | 5 | High |
| Lu and Li (2018) | UN | Henan | Flotation method (NaCl) | 225/360 | ND | 1 | Low |
| Yan (2019) | UN | Hubei | Flotation method (NaCl) | 173/300 | ND | 2 | Middle |
| Cheng et al. (2020) | 2018.08-2019.05 | Henan | Flotation method (NaCl, Suc) | 249/2051 | 134/2051 | 4 | High |
| **East China** |  |  |  |  |  |  |  |
| Lu et al. (1992) | UN | Anhui | Flotation method (NaCl) | 52/105 | ND | 2 | Middle |
| Lu et al. (1994) | 1991.08-1993.07 | Anhui | Flotation method (NaCl) | 100/100 | 4/100 | 3 | Middle |
| Qian and Xu (2003) | UN | Jiangsu | Flotation method (NaCl) | 22/40 | 5/40 | 2 | Middle |
| Wu (2006) | 2005.03 | Jiangsu | Flotation method (NaCl) | 45/100 | 36/100 | 4 | High |
| Zhang et al. (2011) | 2010.03-2010.12 | Fujian | Centrifugal flotation method | 73/1040 | 39/1040 | 4 | High |
| Zhang et al. (2012) | 2009.09-2010.12 | Fujian | Flotation method (NaCl) | 7/30 | ND | 5 | High |
| Zhang et al. (2012) | 2009.09-2010.12 | Jiangsu | Flotation method (NaCl) | 6/40 | ND | 5 | High |
| Zhang et al. (2012) | 2009.09-2010.12 | Jiangxi | Flotation method (NaCl) | 1/20 | ND | 5 | High |
| Zhang et al. (2012) | 2009.09-2010.12 | Shandong | Flotation method (NaCl) | 22/135 | ND | 5 | High |
| Zhang et al. (2012) | 2009.09-2010.12 | Shanghai | Flotation method (NaCl) | 2/20 | ND | 5 | High |
| Zhang et al. (2012) | 2009.09-2010.12 | Zhejiang | Flotation method (NaCl) | 13/40 | ND | 5 | High |
| Zhu and Yuan (2012) | UN | Jiangxi | Flotation method (NaCl) | 240/1301 | ND | 2 | Middle |
| Wen et al. (2014) | 2012.04-2012.10 | Fujian | Others (centrifugal precipitation) | 212/2173 | 212/2173 | 4 | High |
| Liu (2015) | 2014.05-2015.04 | Jiangsu | Flotation method (NaCl, Suc) | 804/2564 | ND | 3 | Middle |
| Chen et al. (2016) | 2014.10-2014.12 | Anhui | Others (Flotation method (Suc)/washed precipitation method/lugol's iodine staining) | 33/500 | ND | 4 | High |
| Sheng et al. (2017) | 2014.10-2014.12 | Anhui | Others (direct smear method/Nest PCR method) | 66/500 | 66/500 | 4 | High |
| Lu and Li (2018) | UN | Jiangxi | Flotation method (NaCl) | 1/10 | ND | 1 | Low |
| Lu and Li (2018) | UN | Shandong | Flotation method (NaCl) | 6/47 | ND | 1 | Low |
| Lu and Li (2018) | UN | Zhejiang | Flotation method (NaCl) | 58/266 | ND | 1 | Low |
| Zhang et al. (2018) | 2016.05-2017.08 | Jiangsu | Others (Flotation method (NaCl)/ Flotation method (Suc)) | 83/1206 | ND | 3 | Middle |
| Yuan et al. (2019) | UN | Jiangsu | Others (Flotation method (NaCl)/washed precipitation method/lugol's iodine staining) | 30/895 | ND | 2 | Middle |
| **North China** |  |  |  |  |  |  |  |
| Fang (2019) | 2018.01-2018.09 | Hebei | Flotation method (NaCl, Suc) | 91/230 | 91/230 | 3 | Middle |
| **Northeast China** |  |  |  |  |  |  |  |
| Yu (2008) | UN | Heilongjiang | Flotation method (NaCl) | 482/1200 | ND | 1 | Low |
| Zhang et al. (2012) | 2009.09-2010.12 | Heilongjiang | Flotation method (NaCl) | 6/40 | ND | 5 | High |
| Zhang et al. (2012) | 2009.09-2010.12 | Jilin | Flotation method (NaCl) | 0/20 | ND | 5 | High |
| Zhang et al. (2012) | 2009.09-2010.12 | Liaoning | Flotation method (NaCl) | 11/50 | ND | 5 | High |
| Lu and Li (2018) | UN | Heilongjiang | Flotation method (NaCl) | 24/85 | ND | 1 | Low |
| Lu and Li (2018) | UN | Jilin | Flotation method (NaCl) | 35/40 | ND | 1 | Low |
| Lu and Li (2018) | UN | Jilin | Flotation method (NaCl) | 12/65 | ND | 1 | Low |
| Lu and Li (2018) | UN | Liaoning | Flotation method (NaCl) | 87/157 | ND | 1 | Low |
| **Northwest China** |  |  |  |  |  |  |  |
| Zhang and Quan (1990) | 1988.08-1988.12 | Shaanxi | Flotation method (NaCl) | 153/209 | 12/209 | 2 | Middle |
| Zhang et al. (1994) | 1991.03-1991.06 | Shaanxi | Centrifugal flotation method | 71/88 | 6/88 | 2 | Middle |
| Ding (2015) | 2011.01-2013.01 | Shaanxi | UN | 136/1399 | 51/1339 | 3 | Middle |
| Lu and Li (2018) | UN | Shaanxi | Flotation method (NaCl) | 34/80 | ND | 1 | Low |
| **South China** |  |  |  |  |  |  |  |
| Liu et al. (1980) | 1979.04-1979.07 | Hubei | Flotation method (NaCl) | 58/588 | ND | 3 | Middle |
| Nong et al. (1992) | 1989.09-1991.12 | Guangxi | Flotation method (NaCl) | 179/723 | 24/723 | 3 | Middle |
| Li (1997) | 1995.12-1996.07 | Guangdong | Flotation method (Suc) | 9/156 | 7/72 | 3 | Middle |
| Zhao et al. (1998) | 1996.05-1996.06 | Hubei | Flotation method (NaCl) | 227/480 | ND | 4 | High |
| Yang et al. (1998) | 1991 | Guangdong | Flotation method (NaCl) | 5/70 | 5/70 | 4 | High |
| Chen et al. (2007) | 2005.07-2006.08 | Guangdong | Flotation method (NaCl) | 58/766 | ND | 5 | High |
| Tang et al. (2007) | UN | Hunan | Flotation method (NaCl) | 321/1301 | ND | 1 | Low |
| Huang et al. (2007) | 2005.05-2005.10 | Hunan | Others (Flotation method) | 256/1230 | ND | 2 | Middle |
| Zhang et al. (2012) | 2009.09-2010.12 | Guangdong | Flotation method (NaCl) | 6/40 | ND | 5 | High |
| Zhang et al. (2012) | 2009.09-2010.12 | Guangxi | Flotation method (NaCl) | 7/30 | ND | 5 | High |
| Zhang et al. (2012) | 2009.09-2010.12 | Hainan | Flotation method (NaCl) | 2/20 | ND | 5 | High |
| Zhang et al. (2012) | 2009.09-2010.12 | Hubei | Flotation method (NaCl) | 2/38 | ND | 5 | High |
| Zhang et al. (2012) | 2009.09-2010.12 | Hunan | Flotation method (NaCl) | 1/20 | ND | 5 | High |
| Li and Liu (2013) | 2011.07-2011.12 | Hunan | Flotation method (NaCl, Suc) | 465/1645 | 322/1645 | 5 | High |
| Tang et al. (2013) | UN | Guangxi | Flotation method (Suc) | 773/3021 | ND | 2 | Middle |
| Li and Lu (2017) | 2016.05-2016.11 | Guangdong | Direct smear method | 114/2437 | ND | 5 | High |
| Lu and Li (2018) | UN | Guangdong | Flotation method (NaCl) | 61/116 | ND | 1 | Low |
| Lu and Li (2018) | UN | Guangxi | Flotation method (NaCl) | 4/35 | ND | 1 | Low |
| **Southwest China** |  |  |  |  |  |  |  |
| Zuo et al. (1987) | 1985.04-1985.10 | Yunnan | Centrifugal flotation method | 441/523 | 49/523 | 2 | Middle |
| Yang and Jiang (2000) | UN | Sichuan | Flotation method (NaCl) | 34/180 | ND | 1 | Low |
| Yang et al. (2004) | 2002.07-2002.09 | Sichuan | Flotation method (NaCl, Suc) | 107/422 | 79/422 | 2 | Middle |
| Nong et al. (2009) | UN | Sichuan | Others (direct smear method /  Flotation method (NaCl)/  teleman method) | 77/261 | ND | 1 | Low |
| Miu and Hu (2008) | 2007.04 | Yunnan | Centrifugal flotation method | 28/50 | 16/50 | 3 | Middle |
| Jiang et al. (2008) | 2007.04-2007.11 | Chongqing | Flotation method (NaCl) | 106/2165 | ND | 4 | High |
| Lai et al. (2011) | 2007-2009 | Chongqing | Flotation method (NaCl) | 640/2971 | 149/2971 | 4 | High |
| Zhang et al. (2012) | 2009.09-2010.12 | Chongqing | Flotation method (NaCl) | 6/35 | ND | 5 | High |
| Zhang et al. (2012) | 2009.09-2010.12 | Sichuan | Flotation method (NaCl) | 9/50 | ND | 5 | High |
| Luo et al. (2013) | 2011.09-2012.08 | Chongqing | Flotation method (NaCl) | 157/515 | 10/246 | 3 | Middle |
| Chen et al. (2015) | 2015.06 | Tibet | Flotation method (NaCl, Suc) | 409/720 | 41/409 | 3 | Middle |

UN*: unclear.

ND*: No data.

Flotation method (NaCl)*: sodium chloride-saturated flotation method.

Flotation method (Suc)*: sugar-saturated flotation method.

Flotation method (NaCl, Suc) *: sugar-saturated sodium chloride solution.

**Table S6.** Included studies and quality scores.

|  | **Reference ID** | **No. tested** | **No. positive** | **Prevalence** | **Random sampling or not** | **Sampled method detailly or not** | **Sample time clearly or not** | **Detection method clearly or not** | **Four or more risk factors or not** | **Score** | **Study Quality** |
| --- | --- | --- | --- | --- | --- | --- | --- | --- | --- | --- | --- |
| 1 | Lai et al.2011 | 2971 | 640 | 21.54% | Y* | N* | Y | Y | Y | 4 | High |
| 2 | Zhang et al.2012 | 779 | 130 | 16.69% | Y | Y | Y | Y | Y | 5 | High |
| 3 | Nong et al.2009 | 261 | 77 | 29.50% | N | N | N | Y | N | 1 | Low |
| 4 | Sheng et al.2017 | 500 | 66 | 13.20% | Y | N | Y | Y | Y | 4 | High |
| 5 | Lu 1994 | 100 | 100 | 100.00% | Y | N | Y | Y | N | 3 | Middle |
| 6 | Chen et al.2016 | 500 | 33 | 6.60% | Y | N | Y | Y | Y | 4 | High |
| 7 | Lu et al.1992 | 105 | 52 | 49.52% | Y | N | N | Y | N | 2 | Middle |
| 8 | Chen et al.2007 | 766 | 58 | 7.57% | Y | Y | Y | Y | Y | 5 | High |
| 9 | Li and Lu 2017 | 2437 | 114 | 4.68% | Y | Y | Y | Y | Y | 5 | High |
| 10 | Nong et al.1992 | 723 | 179 | 24.76% | N | N | Y | Y | Y | 3 | Middle |
| 11 | Zhang et al.2011 | 1040 | 73 | 7.02% | N | Y | Y | Y | Y | 4 | High |
| 12 | Jian et al.2005 | 262 | 23 | 8.78% | N | Y | Y | Y | Y | 4 | High |
| 13 | Chou et al.2008 | 1119 | 467 | 41.73% | Y | Y | Y | N | Y | 4 | High |
| 14 | Liu and Wang 2013 | 1300 | 527 | 40.54% | Y | Y | Y | Y | Y | 5 | High |
| 15 | Han et al.2011 | 1210 | 421 | 34.79% | N | Y | N | Y | Y | 3 | Middle |
| 16 | Zhou et al.1996 | 394 | 171 | 43.40% | N | Y | N | Y | Y | 3 | Middle |
| 17 | Liu et al.1988 | 140 | 41 | 29.29% | Y | N | N | N | N | 1 | Low |
| 18 | Zhao et al.1998 | 480 | 227 | 47.29% | Y | Y | Y | N | Y | 4 | High |
| 19 | Liu et al.1980 | 588 | 58 | 9.86% | N | Y | Y | Y | N | 3 | Middle |
| 20 | Tang et al.2007 | 1301 | 321 | 24.67% | N | N | N | Y | N | 1 | Low |
| 21 | Huang et al.2007 | 1230 | 256 | 20.81% | N | N | Y | N | Y | 2 | Middle |
| 22 | Zhu and Yuan 2012 | 1301 | 240 | 18.45% | Y | N | N | Y | N | 2 | Middle |
| 23 | Wu 2006 | 100 | 45 | 45.00% | Y | N | Y | Y | Y | 4 | High |
| 24 | Lu and Li 2018 | 1261 | 547 | 43.38% | N | N | N | Y | N | 1 | Low |
| 25 | Luo et al.2013 | 515 | 157 | 30.49% | N | Y | Y | Y | N | 3 | Middle |
| 26 | Wen et al.2014 | 2173 | 212 | 9.76% | Y | Y | Y | Y | N | 4 | High |
| 27 | Zhang and Quan 1990 | 209 | 153 | 73.21% | N | N | Y | Y | N | 2 | Middle |
| 28 | Zhang et al.1994 | 88 | 71 | 80.68% | N | N | Y | Y | N | 2 | Middle |
| 29 | Yang et al.2004 | 422 | 107 | 25.36% | N | N | Y | Y | N | 2 | Middle |
| 30 | Cheng et al. 2020 | 2051 | 249 | 12.14% | N | Y | Y | Y | Y | 4 | High |
| 31 | Li and Liu 2013 | 1645 | 465 | 28.27% | Y | Y | Y | Y | Y | 5 | High |
| 32 | Yang et al.1998 | 70 | 5 | 7.14% | N | Y | Y | Y | Y | 4 | High |
| 33 | Liu 2015 | 2564 | 804 | 31.36% | N | Y | Y | Y | N | 3 | Middle |
| 34 | Yuan et al.2019 | 895 | 30 | 3.35% | Y | N | N | Y | N | 2 | Middle |
| 35 | Yang and Jiang 2000 | 180 | 34 | 18.89% | Y | N | N | N | Y | 2 | Middle |
| 36 | Zuo et al.1987 | 523 | 441 | 84.32% | N | N | Y | Y | N | 2 | Middle |
| 37 | Miu and Hu 2008 | 50 | 28 | 56.00% | N | N | Y | Y | Y | 3 | Middle |
| 38 | Yu 2008 | 1200 | 482 | 40.17% | N | N | N | Y | N | 1 | Low |
| 39 | Zhang et al.2008 | 750 | 150 | 20.00% | N | Y | Y | Y | Y | 4 | High |
| 40 | Yan 2019 | 300 | 173 | 57.67% | N | N | N | Y | Y | 2 | Middle |
| 41 | Fang 2019 | 230 | 91 | 39.57% | N | N | Y | Y | Y | 3 | Middle |
| 42 | Ning 2010 | 2388 | 313 | 13.11% | N | Y | Y | Y | Y | 4 | High |
| 43 | Zhao 2005 | 1158 | 100 | 8.64% | N | Y | Y | Y | Y | 4 | High |
| 44 | Ding 2015 | 1399 | 136 | 9.72% | N | N | Y | Y | Y | 3 | Middle |
| 45 | Tang et al.2013 | 3021 | 773 | 25.59% | Y | N | N | Y | N | 2 | Middle |
| 46 | Zhang et al.2018 | 1206 | 83 | 6.88% | N | N | Y | Y | Y | 3 | Middle |
| 47 | Li 1997 | 156 | 9 | 5.77% | N | N | Y | Y | Y | 3 | Middle |
| 48 | Jiang et al.2008 | 2165 | 106 | 4.90% | Y | N | Y | Y | Y | 4 | High |
| 49 | Qian and Xu 2003 | 40 | 22 | 55.00% | N | N | N | Y | Y | 2 | Middle |
| 50 | Chen et al.2015 | 720 | 409 | 56.81% | N | Y | Y | N | Y | 3 | Middle |

Y*: Yes; N*: No.

**References**

1. Lai, M., Zhou, R.Q., Huang, H.C., Hu, S.J., 2011. Prevalence and risk factors associated with intestinal parasites in pigs in Chongqing. China Res Vet Sci. 91, 121-124. doi: 10.1016/j.rvsc.2011.01.025. (In Chinese)
2. Zhou,Y.Q., Hu, M., Zhao, J.L., 2012. Prevalence of coccidian infection in suckling piglets in China. Vet Parasitol. 190, 51-55. doi: 10.1016/j.vetpar.2012.05.015
3. Nong, X., Liang, Z., Song, .LZ., Zhang, L., Zhang, Y.N., Wu, H., Qin, K.Y., 2009. Comparative study on three different methods detecting oocysts of piglet coccidiosis.
4. Sheng, Z.X., Wang, K., Tang, C.C., Gu, Y.F., Li, W.C., 2017. Epidemiological investigation of in large-scale pig farms in Anhui province. Chin J Prev Vet Med. 39, 678-681. doi: 10.3969/j.issn.1008-0425.2017.08.18. (In Chinese)
5. Lu, G., 1994. Investigation on the species of domestic coccidia in some areas of Anhui province. Chin J Anim Infect Dis. 49, 52-53. (In Chinese)
6. Chen, H.L., Wang, K., Liu, H.H., Gu, Y.F., Li, W.C., 2016. Investigation on the infection of intestinal parasites in some large-scale pig farms in Anhui province. Anim Livest Vet Med 48, 98-101. (In Chinese)
7. Lu, F.L., Li, P.Y., Wang, S.C., Ding, S.G., Jiang, H., Zhao, Z.Y., Ma, X.D., 1992. Investigation report on pig coccidia in Huangpi district, Yixian county, Anhui province. Shanghai J Anim Livest Vet Med. 37, 20-21. (In Chinese)
8. Chen, H.L., Lan, T., Chen, X.Q., Lin, R.Q., Liu, M.Y., Weng, Y.B., 2007. Preliminary investigation on coccidiosis infection in piglets in Guangdong intensive pig farms. Swine Prod. 34, 39-40. doi:10.13257/j.cnki.21-1104/s.2007.01.023. (In Chinese)
9. Li, J., Lu, P.Y., 2017. Survey of parasites infection in pigs of intensive farms in Guangdong state farms. Guangdong Agr Sci. 44,132-136. doi:10.16768/j.issn.1004-874X.2017.05.021 (In Chinese)
10. Nong, H.B., Yang, N.H., Teng, B.Z., Chen, F.Q., Chao, Y.L., 1992. Species and epidemiological survey of coccidia in Guangxi. Guangxi Agri Sci. 56, 231-233. (In Chinese)
11. Zhang, S.Z., Jiang, B., Wu, S.H., Lin, L., 2011. Survey of coccidia infection in some large-scale pig farms in Fujian province. Swine Prod. 34, 89-91 doi:10.13257/j.cnki.21-1104/s.2011.02.047 (In Chinese)
12. Jian, F.C., Liu, G.H., Zhao, J.F., Zang, W.M., Shen, C.S., Zhang, L.X., Xue, W., 2005. Epidemiological survey of coccidia in piglets in parts of Henan province. China Anim Livest Vet Med. 32, 50-51. (In Chinese)
13. Qiu, S.X., Guan, F.C., Ren, G.Z., Ning, C.S., Lu, X.Y., Shi, K., Zhang, L.X., 2008. Investigation on intestinal parasite infection in large-scale pig farms in Henan province. Anim Livest Vet Med. 40, 39-42. (In Chinese)
14. Liu, X.L., Wang, L., 2013. Investigation and prevention and control model of parasitic diseases in large-scale pig farms in Henan province. Heilongjiang Anim Sci Vet Med. 62, 124-126. doi:10.13881/j.cnki.hljxmsy.2013.21.049. (In Chinese)
15. Han, Z.Q., Liu, C.C., Gong, Z.X., 2011. Investigation on infection in pigs in Henan province. Anim Livest Vet Med. 43, 77-79. (In Chinese)
16. Zhou, J.X., Hou, A.Z., Zhang, L., Wang, S.R., Wang, T.Q., Feng, X.R., 1996. Species and epidemiological survey of pig Coccidia in Henan province. Chin J Anim Infect Dis. 27, 41-42. (In Chinese)
17. Liu, W.Z., Li, D.Y., Li, Z.L., 1988. Preliminary report on the investigation of species of pig coccidia in Henan province. J Henan Agr Sci. 48, 29-30. doi:10.15933/j.cnki.1004-3268.1988.02.015. (In Chinese)
18. Zhao, J.L., He, G.S., Yao, B.A., Xia, X.S., Wang, X., Ma, L.H., 1998. Study on comprehensive prevention and control measures of parasitic diseases in large-scale pig farms in Hubei province I. Investigation on parasitic infections. Hubei Agr Sci. 44:51-53 doi:10.3969/j.issn.0439-8114.1998.04.019 (In Chinese)
19. Liu, Z.L., Wang, H.J., Zhang, S.X., Zeng, X.G., Chen, X.Q., Ge, R.W., Ma, L.H., Chen, Y.Z., 1980. Investigation report on pig parasites in Hubei Province. Hubei J Anim Vet Sci. 1, 10,24-27. doi:10.16733/j.cnki.issn1007-273x.1980.01.006. (In Chinese)
20. Tang, L.D., Tang, H.W., Liu, Y., 2007. Investigation on gastrointestinal parasite infection in large-scale pig farms in Hunan province. Hubei J Anim Vet Sci 34, 45-46 doi:10.13257/j.cnki.21-1104/s.2007.02.029. (In Chinese)
21. Huang, J.H., Tang, W., Tan, K.Q., 2007. Survey of intestinal parasitic infections in large-scale pig farms in Yongzhou area, Hunan Province. Chin J Vet Med. 67, 30-31. doi:10.3969/j.issn.0529-6005.2007.06.013. (In Chinese)
22. Zhu, Q.L., Yuan, G.M., 2012. Epidemiological survey of major intestinal parasitic diseases in intensive pig farms in some cities of Jiangxi. Chin J Vet Med. 38, 15-17. (In Chinese)
23. Wu, J.B., 2006. Investigation on the species and infection of pig coccidia in Nanchang area. Chin J Anim Infect Dis. 14, 19-21. doi:10.3969/j.issn.1674-6422.2006.02.006. (In Chinese)
24. Lu, S.S., Li, Q.R., 2018. Epidemiological survey of coccidiosis in piglets nationwide. Chin J Anim Infect Dis. 13, 52-54. doi:10.16174/ j.cnki.115435.2018.12.010. (In Chinese)
25. Luo, P.F., Wei, Z.P., Zhou, R.Q., 2013. Epidemiological survey of swine coccidiosis and the species identification of coccidia in Rongchang county. Heilongjiang Anim Sci Vet Med. 62, 94-96. doi:10.13881/j.cnki.hljxmsy.2013.15.053. (In Chinese)
26. Wen, F.L., Yue, L.P., Huang, C.Q., Zhao, D.Y., Wang, K., Chen, R.F., Hu, L.Y., Wang, S.K., 2014. Investigation and analysis of coccidiosis infection in pigs in some pig farms in Fujian. Anim Livest Vet Med. 46: 69-74. (In Chinese)
27. hang, B.X., Quan, Z.H., 1990. Study on the species, distribution and infection status of pig coccidia in Shaanxi province. J Anim Sci Vet Med. 9, 18-22. (In Chinese)
28. Zhang, J.L., Yu, S.K., Feng, Y., Li, Y.J., 1994. Investigation on the pathogens of pig coccidia in yangling district of Shaanxi province and description of a New Species. Chin J Vet Sci. 14, 271-274. doi:10.16303/j.cnki.1005-4545.1994.03.017. (In Chinese)
29. Yang, G.Y., Zeng, Z.Y., Guo, L., Xu, Y.H., Wang, S.X., Chen, Y., Lan, H., Li, Q.H., 2004. Epidemiological survey of coccidiosis in piglets in Sichuan province. Chin Vet Sci. 34, 31-34. doi:10.16656/j.issn.1673-4696.2004.03.007. (In Chinese)
30. Cheng, J.Z., Liu, G.H., Yan, R.Q., Zhao, S.J., Fang, D.X., 2020. Epidemiological investigation and analysis of swine coccidiosis in Henan Province. Swine Prod. 35, 94-97. doi: 10.13257/j.cnki.21-1104/s.2020.01.036. (In Chinese).
31. Li, W.H., Liu, Y., 2013. Investigation on the infection of pig coccidia in pig farms in Xintian county. Hunan J Animal Sci Vet Med. 41, 26-28. doi:10.3969/j.issn.1006-4907.2013.01.011 (In Chinese)
32. Yang, L.J., Gao, Y.Q., Li, G.X., 1998. Identification of isopods infected by piglets. Chin J Anim Infect Dis. 6,19-20. (In Chinese)
33. Liu, L., 2015. Epidemiological investigation and analysis of coccidiosis in pig farms in Rudong county, Jiangsu province. Nongjia Keji. 31, 111-112. (In Chinese)
34. Yuan, C., Zhang, B.C., Dai, L.H., Zhao, H.K., Xu, B., Gan, J.T., Wei, D.X., 2019. Investigation of infection by intestinal parasites in some large-scale pig farms in the mid-Jiangsu area. Anim Livest Vet Med. 51, 76-79. (In Chinese)
35. Yang, G.Y., Jiang, Z.M., 2000. Investigation and study on pig parasites in a large-scale pig farm in Sichuan. Livest Poultry Ind. 30, 36-37. (In Chinese)
36. Zuo, Y.X., Chen, F.Q., Song, X.L., Li, S.B., Yuan, Q.M., Wang, K.H., 1987. Investigation on the pathogens of pig coccidia in Yunnan province. Chin Vet Sci. 17, 22-26. (In Chinese)
37. Miu, J.Y., Hu, J.J., 2008. Preliminary investigation on the species of coccidia in Kunming city, Yunnan province. Anim Livest Vet Med. 40, 55-57. (In Chinese)
38. Yu, C.X., 2008. Investigation and prevention of coccidiosis in piglets. Mod Anim Livest. 36, 129-130. (In Chinese)
39. Zhang, W.L., Chen, Y.P., Wang, T.Q., 2008. Investigation on the species and infection of coccidia in large-scale pig farms in Yichuan county. Yunnan J Anim Sci Vet Med. 48, 7-9. (In Chinese)
40. Yan, Y.H., 2019. Investigation and control of pig parasitic diseases in large-scale pig farms in Xianning City. Hubei J Anim Vet Sci. 40:33-34 doi:10.16733/j.cnki.issn1007-273x.2019.05.017. (In Chinese)
41. Fang, X.M., 2019. Investigation and control of coccidiosis in pigs in large-scale pig farms in the Tangshan area. Mod Anim Livest. 40, 44-45.
42. Ning, C.K., 2010. The epidemiological investigation, pathogenicity experiments, pharmic prevention and treatment of *Isospora Suis* in Henan. Dissertation, Nanjing Agricultural College. (In Chinese)
43. Zhao, Z.J., 2005. Investigation on infection of swine coccidium in Luoyang and pathogenicity and treatment of neosporosis in piglets. Dissertation, Jilin Univ. (In Chinese)
44. Ding, L., 2015. Studies on intestinal parasite species and *Cryptosporidium* identification in swine in Shaanxi province. Dissertation, Northwest Agri For Univ. (In Chinese)
45. Tang, L.S., Tao, L., Wei, X.R., Qin, R.P., Wei, Z.F., Huang, D.Y., Lan, J.H., Lan, M.Y., Li, J., Chen, Z.X., Yang, W., Huang, W.Y., 2013. Investigation of intestinal parasite infection in Guangxi pig herd. J South Agri. 44, 516-520. (In Chinese)
46. Zhang, B.C., Yuan, C., Wang, X.B., Sun, Q., Liu, L., Yang, L., Zhu, R., Qi, J.J., Li, J., Liu, P.G., Jiang, C.M., 2018. Investigation of swine intestinal parasite infection in Taizhou. Anim Husbandry Vet Med. 50, 112-116. (In Chinese)
47. Li, X.L., 1997. Investigation of *Isospora* in some pig farms in Guangdong Province. Guangdong J Anim Vet Sci. 22, 25-26. (In Chinese)
48. Jiang, Q., Rao, M., Zhang, L.J., Fan, C.L., Hu, J.L., Lin, R.Q., Zhu, X.Q., 2008. Investigation of intestinal parasite infection in pigs in Rongchang County. Chin J Anim Infect Dis. 16, 26-30. (In Chinese)
49. Qian, X.Z., Xu, Q.Q., 2003. Preliminary investigation of pig coccidia species in Taizhou City, Jiangsu Province. Chin J Anim Infect Dis. 11, 31-32. (In Chinese)
50. Chen, X.Y., Xia, C.Y., Lan, L., Wang, Y.J., Ma, J.Y., 2015. Epidemiological survey and species identification of Tibetan pig coccidiosis along Lalin Highway. Swine Prod. 30, 107-108. doi: 10.13257/j.cnki.21-1104/s.2015.05.048. (In Chinese)

**Table S7.** Egger’s for Publication Bias.

| slope | bias | se. bias | t | df | p-value |
| --- | --- | --- | --- | --- | --- |
| 0.032 | -20.987 | 2.167 | -9.684 | 48 | 7.166e - 13 |

**Table S8.** Pooled prevalence of different coccidia species in China.

| Species of *Coccidia* | No.  studies | No.  tested | No.  positive | % (95% CI) |
| --- | --- | --- | --- | --- |
| *C. suis* | 26 | 20470 | 1834 | 9.1% (6.9-11.9) |
| *E. cerdonis* | 6 | 1411 | 196 | 15.1% (6.1-37.1) |
| *E. debliecki* | 13 | 4338 | 589 | 16.8% (10.5-27.0) |
| *E. neodebliecki* | 9 | 2395 | 500 | 21.1% (13.0-34.5) |
| *E. perminuta* | 12 | 3657 | 398 | 11.9% (7.4-19.2) |
| *E. polita* | 5 | 1852 | 392 | 14.1% (5.8-34.2) |
| *E. porci* | 9 | 2732 | 416 | 14.6% (10.6-20.1) |
| *E. scabra* | 13 | 3707 | 751 | 18.9% (13.4-26.6) |
| *E. spinosa* | 6 | 2362 | 272 | 8.6% (5.2-14.2) |
| *E. suis* | 14 | 4747 | 935 | 19.0% (12.6-28.7) |
| *E. yanglingensis* | 2 | 175 | 15 | 9.9% (3.1-32.0) |

**Table S9.** List of abbreviations.

| CNKI | Chinese Web of knowledge |
| --- | --- |
| WTO | World Trade Organization |
| PRISMA | Preferred Reporting Items for Systematic Reviews and Meta-Analyses |
| GRADE | Grading of Recommendations Assessment, Development, and Evaluation |
| *E. debliecki* | *Eimeria deblieck* |
| *E. neodebliecki* | *Eimeria neodebliecki* |
| *E. perminuta* | *Eimeria perminuta* |
| *E. polita* | *Eimeria polita* |
| *E. porci* | *Eimeria porci* |
| *E. scabra* | *Eimeria scabra* |
| *E. suis* | *Eimeria suis* |
| *E. spinosa* | *Eimeria spinosa* |
| *E. cerdonis* | *Eimeria cerdonis* |
| *E. yanglingensis* | *Eimeria yanglingensis* |
| *C. almaataensis* | *Cystoisospor almaataensis* |
| *C. neyrai* | *Cystoisospor neyrai* |
| *C. sundar-banensis* | *Cystoisospor sundar-banensis* |
| *C. suis* | *Cystoisospora suis* |
