# Supplementary material for: Prevalence of coccidia in domestic pigs in China between 1980 and 2019: a systematic review and meta-analysis
Source: Parasit Vectors. 2021 May 10;14:248. doi: 10.1186/s13071-021-04611-x (PMC8108339; doi:10.1186/s13071-021-04611-x)
Supplement: Supplementary file 2 — Additional file 2: Figure S1. Funnel plot with pseudo 95% confidence intervals used in the assessment of publication bias. Figure S2. Assessment of publication bias using Egger’s test. Figure S3. Sensitivity analysis. Figure S4. Multivariate meta-regression analysis by publication year. Funnel plot with pseudo 95% confidence intervals used in the assessment of publication bias (C. suis). Assessment of publication bias (C. suis) using Egger’s test. Sensitivity analysis (C. suis). Figure S5. Multivariate meta-regression analysis by publication year (C. suis). [file 13071_2021_4611_MOESM2_ESM.pptx]

## Slide 1
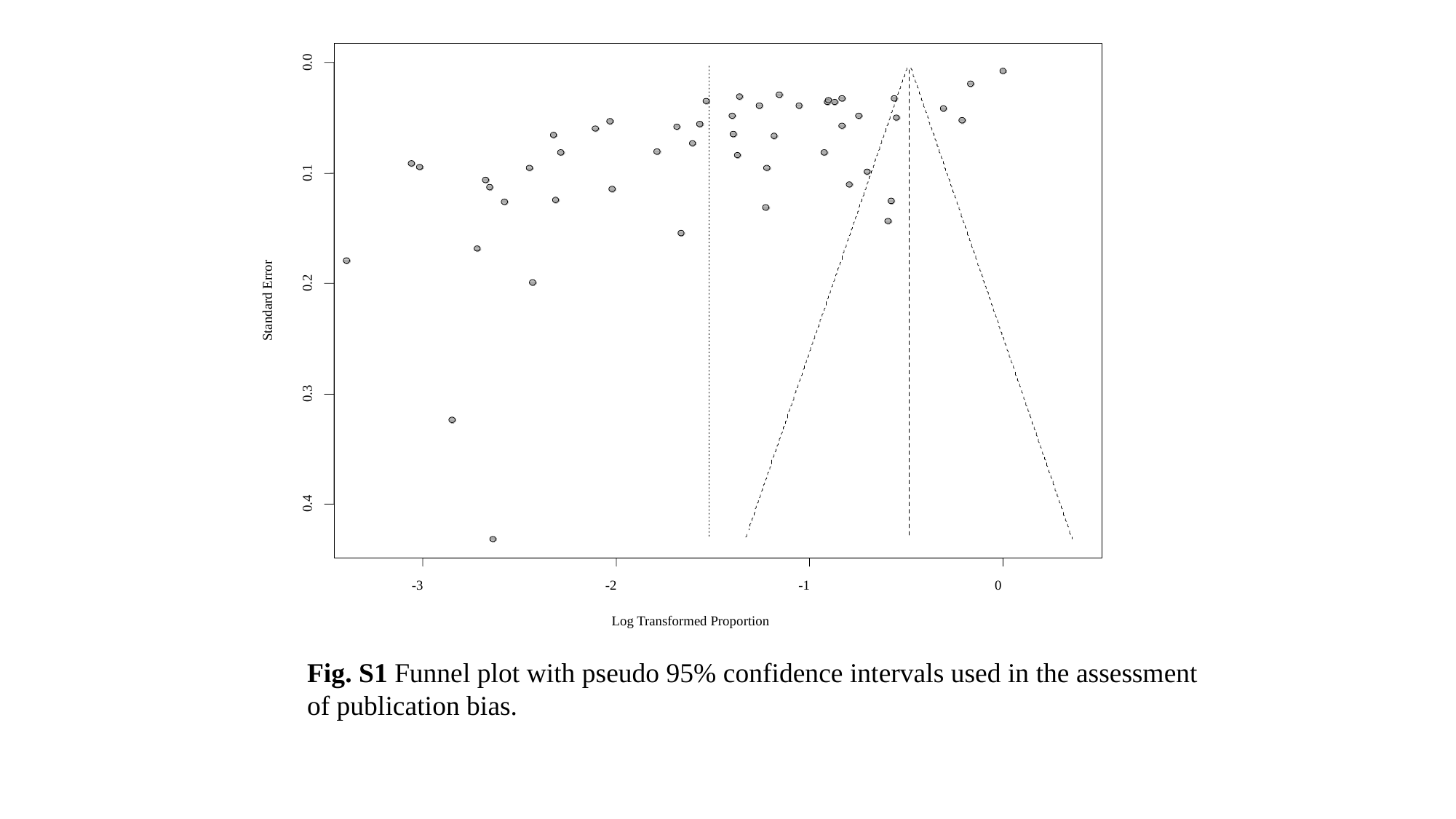

0.0
0.1
0.2
Standard Error
0.3
0.4
-3
-2
-1
0
Log Transformed Proportion
Fig. S1 Funnel plot with pseudo 95% confidence intervals used in the assessment of publication bias.

## Slide 2
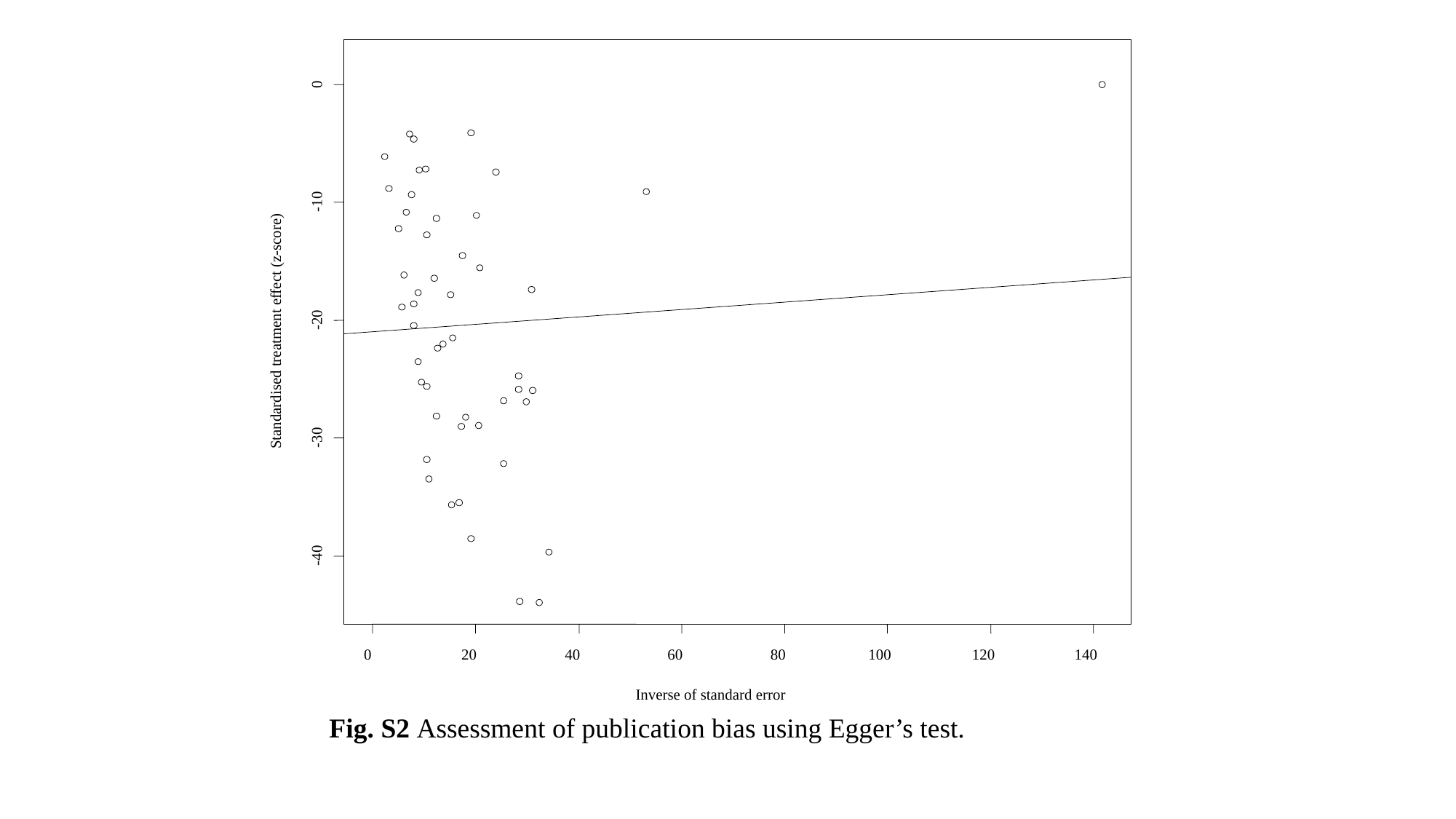

0
-10
-20
Standardised treatment effect (z-score)
-30
-40
0
20
40
60
80
100
120
140
Inverse of standard error
Fig. S2 Assessment of publication bias using Egger’s test.

## Slide 3
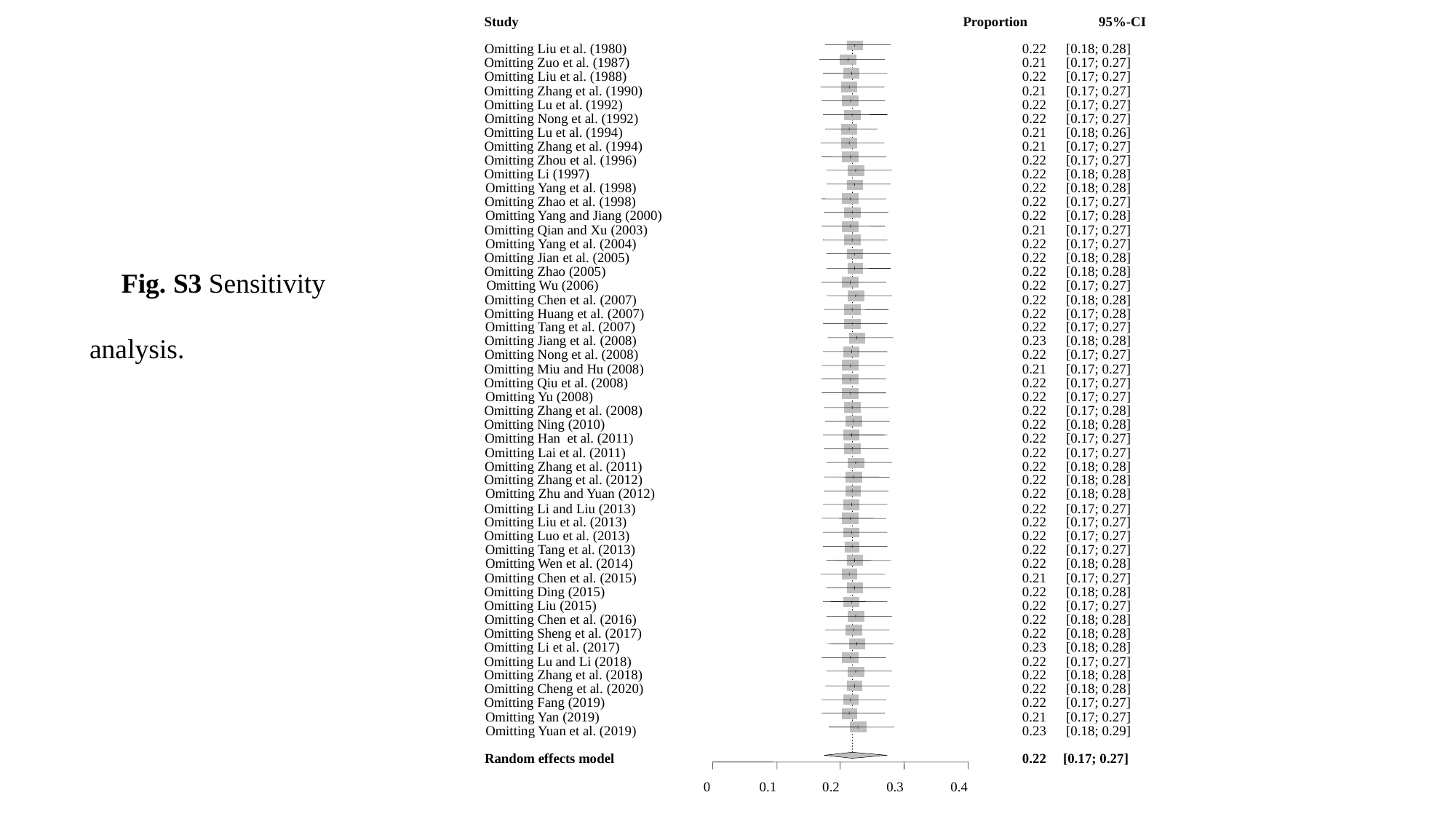

Study
Omitting Liu et al. (1980)
Omitting Zuo et al. (1987)
Omitting Liu et al. (1988)
Omitting Zhang et al. (1990)
Omitting Lu et al. (1992)
Omitting Nong et al. (1992)
Omitting Lu et al. (1994)
Omitting Zhang et al. (1994)
Omitting Zhou et al. (1996)
Omitting Li (1997)
Omitting Yang et al. (1998)
Omitting Zhao et al. (1998)
Omitting Yang and Jiang (2000)
Omitting Qian and Xu (2003)
Omitting Yang et al. (2004)
Omitting Jian et al. (2005)
Omitting Zhao (2005)
Omitting Wu (2006)
Omitting Chen et al. (2007)
Omitting Huang et al. (2007)
Omitting Tang et al. (2007)
Omitting Jiang et al. (2008)
Omitting Nong et al. (2008)
Omitting Miu and Hu (2008)
Omitting Qiu et al. (2008)
Omitting Yu (2008)
Omitting Zhang et al. (2008)
Omitting Ning (2010)
Omitting Han et al. (2011)
Omitting Lai et al. (2011)
Omitting Zhang et al. (2011)
Omitting Zhang et al. (2012)
Omitting Zhu and Yuan (2012)
Omitting Li and Liu (2013)
Omitting Liu et al. (2013)
Omitting Luo et al. (2013)
Omitting Tang et al. (2013)
Omitting Wen et al. (2014)
Omitting Chen et al. (2015)
Omitting Ding (2015)
Omitting Liu (2015)
Omitting Chen et al. (2016)
Omitting Sheng et al. (2017)
Omitting Li et al. (2017)
Omitting Lu and Li (2018)
Omitting Zhang et al. (2018)
Omitting Cheng et al. (2020)
Omitting Fang (2019)
Omitting Yan (2019)
Omitting Yuan et al. (2019)
Random effects model
0
0.1
0.2
0.3
0.4
Proportion
95%-CI
0.22
[0.18; 0.28]
0.21
[0.17; 0.27]
0.22
[0.17; 0.27]
0.21
[0.17; 0.27]
0.22
[0.17; 0.27]
0.22
[0.17; 0.27]
0.21
[0.18; 0.26]
0.21
[0.17; 0.27]
0.22
[0.17; 0.27]
0.22
[0.18; 0.28]
0.22
[0.18; 0.28]
0.22
[0.17; 0.27]
0.22
[0.17; 0.28]
0.21
[0.17; 0.27]
0.22
[0.17; 0.27]
0.22
[0.18; 0.28]
0.22
[0.18; 0.28]
0.22
[0.17; 0.27]
0.22
[0.18; 0.28]
0.22
[0.17; 0.28]
0.22
[0.17; 0.27]
0.23
[0.18; 0.28]
0.22
[0.17; 0.27]
0.21
[0.17; 0.27]
0.22
[0.17; 0.27]
0.22
[0.17; 0.27]
0.22
[0.17; 0.28]
0.22
[0.18; 0.28]
0.22
[0.17; 0.27]
0.22
[0.17; 0.27]
0.22
[0.18; 0.28]
0.22
[0.18; 0.28]
0.22
[0.18; 0.28]
0.22
[0.17; 0.27]
0.22
[0.17; 0.27]
0.22
[0.17; 0.27]
0.22
[0.17; 0.27]
0.22
[0.18; 0.28]
0.21
[0.17; 0.27]
0.22
[0.18; 0.28]
0.22
[0.17; 0.27]
0.22
[0.18; 0.28]
0.22
[0.18; 0.28]
0.23
[0.18; 0.28]
0.22
[0.17; 0.27]
0.22
[0.18; 0.28]
0.22
[0.18; 0.28]
0.22
[0.17; 0.27]
0.21
[0.17; 0.27]
0.23
[0.18; 0.29]
0.22
[0.17; 0.27]
Fig. S3 Sensitivity analysis.

## Slide 4
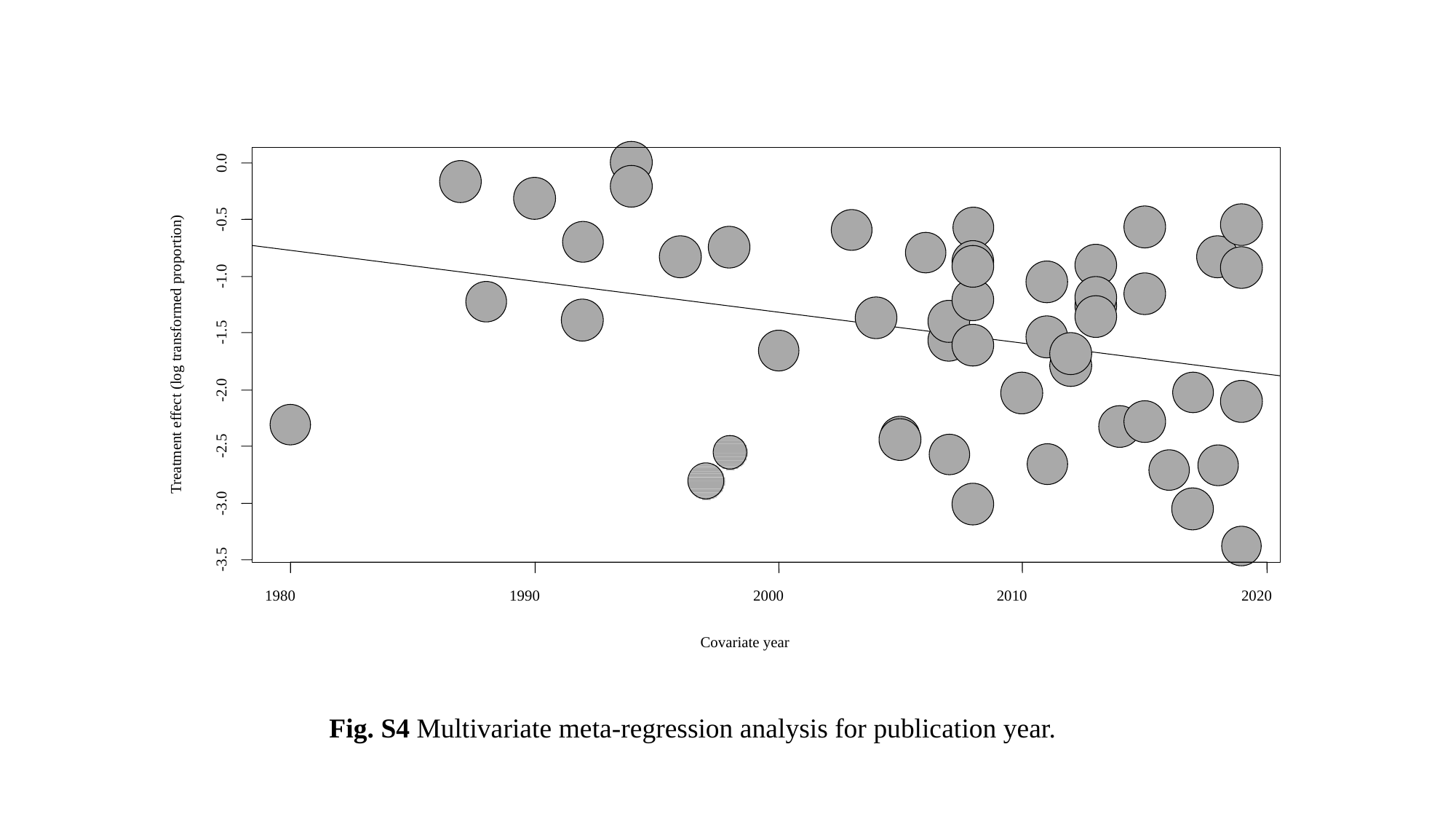

0.0
-0.5
-1.0
-1.5
Treatment effect (log transformed proportion)
-2.0
-2.5
-3.0
-3.5
1980
1990
2000
2010
2020
Covariate year
Fig. S4 Multivariate meta-regression analysis for publication year.

## Slide 5
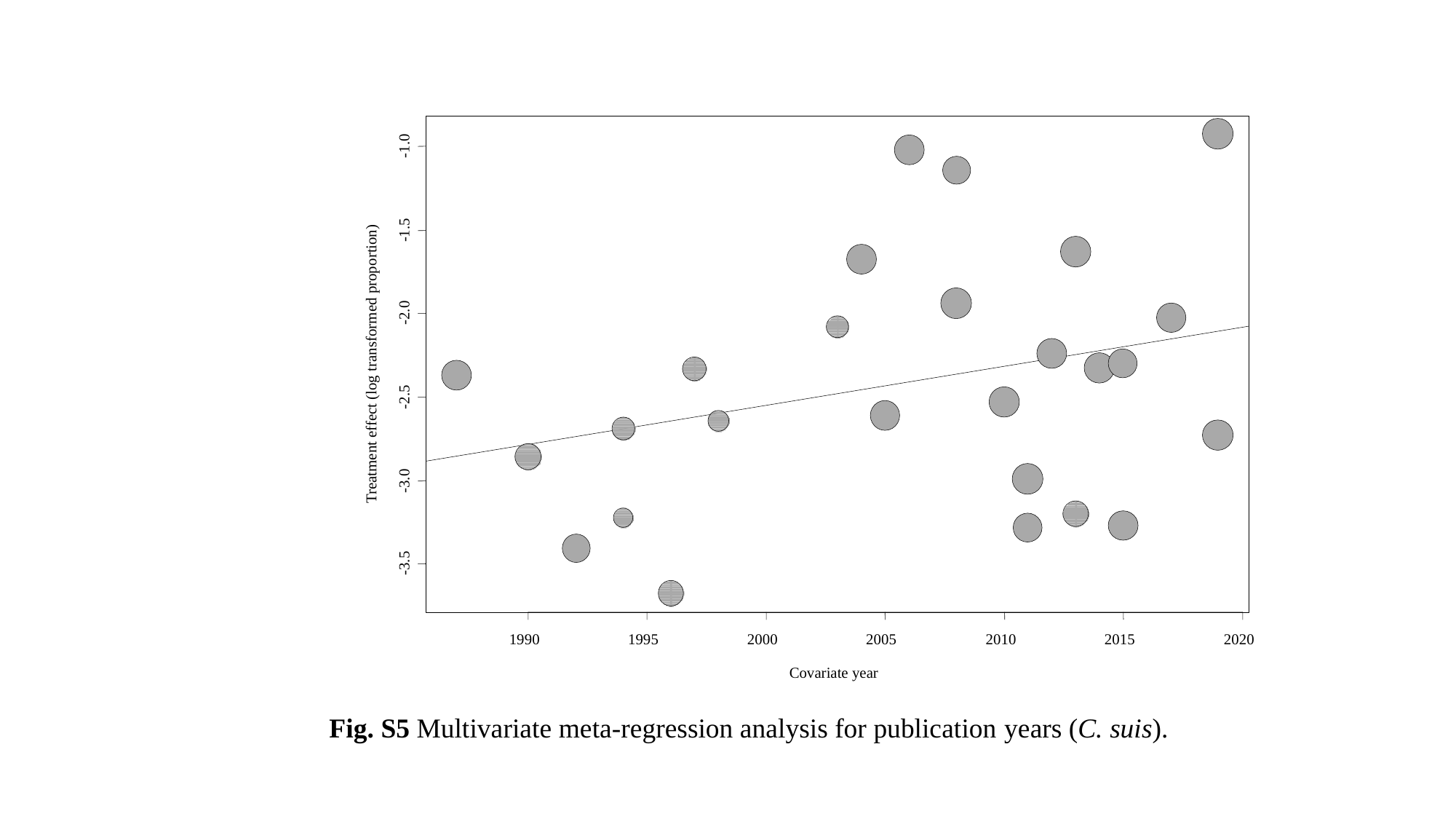

Treatment effect (log transformed proportion)
Covariate year
-1.0
-1.5
-2.0
-2.5
-3.0
-3.5
1990
1995
2000
2005
2010
2015
2020
Fig. S5 Multivariate meta-regression analysis for publication years (C. suis).
